# The interplay of UV and cutaneous papillomavirus infection in skin cancer development

**DOI:** 10.1371/journal.ppat.1006723

**Published:** 2017-11-30

**Authors:** Daniel Hasche, Sonja Stephan, Ilona Braspenning-Wesch, Julita Mikulec, Martina Niebler, Hermann-Josef Gröne, Christa Flechtenmacher, Baki Akgül, Frank Rösl, Sabrina E. Vinzón

**Affiliations:** 1 Division of Viral Transformation Mechanisms, German Cancer Research Center (DKFZ), Heidelberg, Germany; 2 Division of Virus-associated Carcinogenesis, German Cancer Research Center (DKFZ), Heidelberg, Germany; 3 Division of Cellular and Molecular Pathology, German Cancer Research Center (DKFZ), Heidelberg, Germany; 4 Institute of Pathology, University Hospital Heidelberg, Heidelberg, Germany; 5 Institute of Virology, University of Cologne, Cologne, Germany; University of Wisconsin Madison School of Medicine and Public Health, UNITED STATES

## Abstract

Cutaneous human papillomaviruses (HPVs) are considered as cofactors for non-melanoma skin cancer (NMSC) development, especially in association with UVB. Extensively studied transgenic mouse models failed to mimic all aspects of virus-host interactions starting from primary infection to the appearance of a tumor. Using the natural model *Mastomys coucha*, which reflects the human situation in many aspects, we provide the first evidence that only UVB and *Mastomys natalensis* papillomavirus (MnPV) infection strongly promote NMSC formation. Using UVB exposures that correspond to UV indices of different geographical regions, irradiated animals developed either well-differentiated keratinizing squamous cell carcinomas (SCCs), still supporting productive infections with high viral loads and transcriptional activity, or poorly differentiated non-keratinizing SCCs almost lacking MnPV DNA and in turn, early and late viral transcription. Intriguingly, animals with the latter phenotype, however, still showed strong seropositivity, clearly verifying a preceding MnPV infection. Of note, the mere presence of MnPV could induce γH2AX foci, indicating that viral infection without prior UVB exposure can already perturb genome stability of the host cell. Moreover, as shown both under *in vitro* and *in vivo* conditions, MnPV E6/E7 expression also attenuates the excision repair of cyclobutane pyrimidine dimers upon UVB irradiation, suggesting a viral impact on the DNA damage response. While mutations of Ras family members (e.g. *Hras*, *Kras*, and *Nras*) were absent, the majority of SCCs harbored—like in humans—*Trp53* mutations especially at two hot-spots in the DNA-binding domain, resulting in a loss of function that favored tumor dedifferentiation, counter-selective for viral maintenance. Such a constellation provides a reasonable explanation for making continuous viral presence dispensable during skin carcinogenesis as observed in patients with NMSC.

## Author summary

Epidemiological data already strongly suggest an involvement of cutaneous papillomaviruses in the development of NMSC. However, since the viral DNA is frequently lost during progression from precursor lesions to NMSC—which is in contrast to high-risk mucosal HPVs in the context of anogenital cancer—their etiological role is still controversially discussed. Although extensively studied in experimentally infected or transgenic models, studies in this context are still hampered by the lack of suitable preclinical models mimicking all stages as found in humans, starting from primary infection to the appearance of a tumor. Here, we provide the first evidence that only the cooperation between UVB and cutaneous papillomavirus infection strongly favors the development of skin tumors in the natural model *Mastomys coucha*. This study strongly supports the importance of cutaneous papillomaviruses in NMSC development and explains the loss of viral DNA during malignization of UV-induced tumors, a feature commonly observed in human SCCs.

## Introduction

More than 20% of all human cancers have an infectious etiology [1]. In the case of anogenital cancer, high-risk human papillomaviruses (HPVs) of genus alpha (α-HPVs) were identified to be necessary and sufficient to induce cervical cancer [2]. Moreover, although still controversially discussed, there is increasing evidence that infection with certain cutaneous HPVs of genus beta (β-HPVs)—in conjunction with UV exposure—is a crucial factor in the development of non-melanoma skin cancer (NMSC) and, particularly, squamous cell carcinoma (SCC) [3]. NMSC is the most frequent cancer in Caucasians, especially affecting organ transplant recipients (OTR) after systemic immunosuppression [4]. The risk of OTRs to get a SCC increases up to 250-fold upon iatrogenic immunosuppression in comparison to the healthy population [5] and the frequency of tumor formation correlates with the extent and duration of immunosuppression [6]. Although mortality from NMSC is rare in the immunocompetent population, it represents a considerable burden on the health-care system, particularly considering immunocompromised patients [7]. It is estimated that up to 40% of OTRs will develop basal cell carcinomas (BCCs) and SCCs within the first 10 years after transplantation, and up to 80% after 20 years [8]. Consequently, the proof of a causal link between cutaneous PV infection and NMSC would support the concept of a broader vaccination strategy, eliminating at least one important cofactor of skin carcinogenesis [9].

Skepticism about an etiology of cutaneous HPVs in NMSC is mainly based on the finding that SCCs either completely lack HPV DNA or that only a few cells are virus-positive [10], therefore not fulfilling the first Koch postulate pointing toward an infectious causality in tumor development [11]. However, there is current evidence suggesting that cutaneous HPVs could act through a “hit-and-run” mechanism in which viral oncogene expression plays a role in initiation of transformation but is ultimately no longer required for tumor maintenance [12]. Additionally, numerous seroepidemiological reports support a role of certain β-HPVs in NMSC development [13,14] despite their occasional absence within a malignant lesion.

The examination of the interplay between potential tumor viruses and additional risk factors requires appropriate preclinical models that mirror all stages of disease, starting from primary infection to the final manifestation of a tumor. Previously, we used the unique model *Mastomys coucha*, a multimammate rodent, to investigate the role of cutaneous PVs in NMSC formation [15]. These animals are immunocompetent and their skin becomes—similar to HPV in humans—infected early in their lifetime with *Mastomys natalensis* PV (MnPV) [16,17], which lacks the E5 open reading frame, a typical feature of β-HPVs [18]. Lesions can be found all over the body and are not restricted to local areas as reported for *Mus musculus* PV1 (MmuPV1) in mice [19,20]. MnPV is naturally spread within our colony and follow-up studies also allowed us to dissect the complete course of antibody responses during all stages of infection [17,21]. In addition, a virus-free colony enables infections under defined experimental conditions [21].

In a subpopulation of our MnPV-positive colony, animals spontaneously developed benign and, more rarely, malignant skin tumors (e.g. papillomas, keratoacanthomas, SCCs) in an age-dependent manner that are histologically similar to lesions found in patients [15]. Based on this property, we recently provided the proof-of-concept that a MnPV-L1 virus-like-particle (VLP)-based vaccine could completely prevent all forms of tumor formation in these animals even under immunosuppressive conditions as found in OTRs [21].

Since in humans more than 80% of pre-neoplastic skin lesions and NMSCs appear at sun-exposed areas [6], particularly UVB radiation (290–320 nm) is considered as a central risk factor for NMSC development and causes DNA photoproducts, e.g. cyclobutane pyrimidine dimers (CPDs), that predominantly lead to C→T and rarely CC→TT transitions [22]. Physiologically, UVB induces activation of p53 leading to cell cycle arrest and DNA repair or—at higher doses—to apoptosis [23]. However, if p53 function is disturbed, for example by a preceding infection with certain cutaneous HPV types, genetically damaged cells can accumulate, thereby promoting the development of NMSC [24,25]. Although cutaneous HPVs cannot degrade p53 as high risk α-HPV types, their E6 proteins affect many intracellular pathways involved in cell cycle control, DNA repair and maintenance of a normal cellular phenotype [24,26–30].

Additionally, several studies with transgenic mice indicate a mechanistic link between cutaneous HPV and UV exposure in the development of skin tumors [31–33]. However, these mouse models are hampered by the fact that the constitutively expressed transgenes are recognized as self-antigens and therefore only incompletely reflect a natural infection in terms of viral expression, virus production and seroconversion.

Hence, questions still open in the context of NMSC development are the following: (i) how do UV irradiation and viral infection favor the outcome of SCCs in immunocompetent animals; (ii) what kinds of tumors are induced and (iii) are there similarities to the situation in humans with respect to histology, viral loads, serology and genomic signatures?

In our study we used *Mastomys coucha* with a low spontaneous skin tumor rate to directly follow up the interplay of PV infection and UVB in naturally MnPV-infected animals in comparison to their virus-free counterparts. With this preclinical model that mimics the human situation in many aspects, we provide the first evidence that cells naturally infected with cutaneous PVs are prone to NMSC development during chronic UVB exposure.

## Results

### UVB irradiation induces skin tumors only in MnPV-infected *Mastomys coucha*

**Fig 1:**
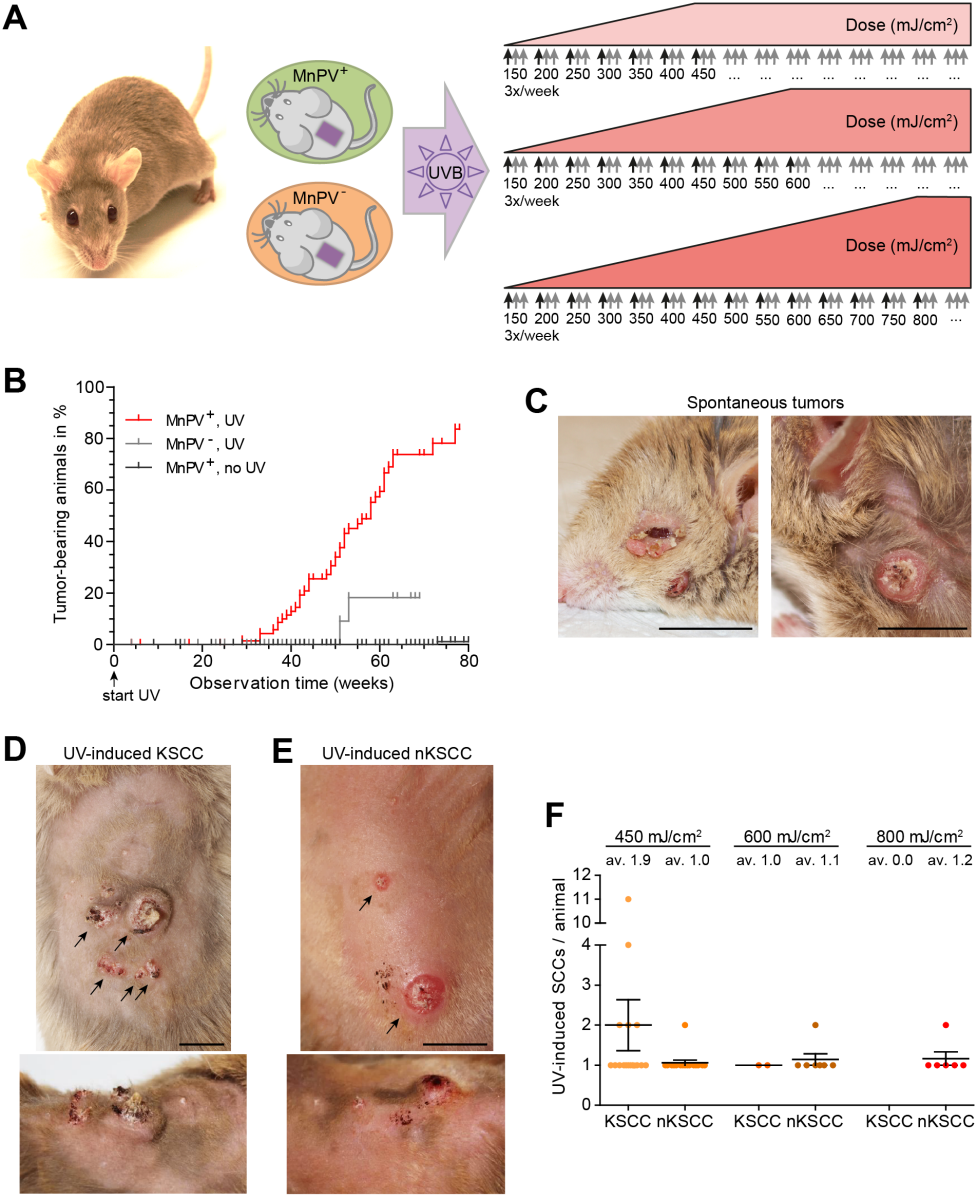
Study design and tumor development. **A)** *Mastomys coucha* as a model for cutaneous papillomavirus infection. In the study, naturally MnPV-infected animals (MnPV^+^) as well as virus-free control animals (MnPV^-^) were irradiated three times per week with UVB. The starting dose of 150 mJ/cm^2^ was increased weekly by 50 mJ/cm^2^ until the desired final dose was reached (450, 600 or 800 mJ/cm^2^, respectively). Black arrows indicate an increase of the dose, gray arrows the subsequent application of this dose. The irradiation was continued until the animals were sacrificed or died. **B)** Kaplan-Meier curves demonstrating the percentage of irradiated virus-infected (MnPV^+^, UV^+^), virus-free (MnPV^-^, UV^+^) and unirradiated virus-infected (MnPV^+^, UV^-^) tumor-bearing animals. **C)** Two examples of spontaneous skin lesions arising in naturally infected animals. **D)** Examples of UV-induced keratinizing SCCs (KSCC) with similarities to human keratoacanthomas. **E)** Examples of UV-induced non-keratinizing SCCs (nKSCC) (C, D and E: scale bars: 10 mm). **F)** Number of KSCCs and nKSCCs in correlation with the final UV doses. Note that KSCCs preferentially appeared at the lowest dose, nKSCCs preferentially at higher doses (Mean ± SEM; animal numbers: see Table 1; av: average number of tumors).

To study the effect of UV exposure, naturally MnPV-infected (MnPV^+^) and MnPV-free (MnPV^-^) animals with an age of 14 weeks were irradiated at the shaved back three times per week with increasing doses of UVB light (Fig 1A). The doses used in our experimental setting were calculated based on information of the World Health Organization [34] and the German Federal Office for Radiation Protection (BfS) [35]. For instance, 450 mJ/cm^2^ UVB (312 nm) corresponds to 6h of sun exposure in Paris, France in May, where a UV index (UVI) of 6.3 is reached. Accordingly, a dose of 600 mJ/cm^2^ is reached after the same time in New York, USA in June (calculated UVI = 8.4) and 800 mJ/cm^2^ in Darwin, Australia in September (calculated UVI = 11.1). Under these conditions, the median time for the onset of tumors was 58 weeks for the MnPV^+^ colony, in which a total of 44 out of 78 animals (56%) developed single or multiple continuously growing skin tumors. Conversely, a median time for tumor development could not be assessed for the MnPV^-^ UV-irradiated (2 out of 37 animals, 5%) and the MnPV^+^ unirradiated colony (1 out of 155 animals, <1%), due to the low tumor incidence of the latter (Table 1).

**Table 1:** Summary of the absolute numbers of tumors in the different groups, percentage of tumor-bearing animals and median time of tumor development.

MnPV status	Dose (mJ/cm^2^)	Animals with tumors during observation time (80 weeks)	Tumor	% of group	% total	Median of tumor occurrence (weeks)
Infected	no UV	1/155	Spontaneous	<1	<1	not assessable
	150–450	31/56	UV-induced	55	56	56
	150–600	7/12		58		58
	150–800	6/10		60		61
Uninfected	150–450	1/19	UV-induced	5	5	not assessable
	150–600	0/8		0		not assessable
	150–800	1/10		10		not assessable

To examine the time dependency of tumor formation, we plotted these results in a Kaplan-Meier curve (Fig 1B), showing that in the MnPV^+^ colony the first UV-induced skin lesions appeared after approximately 30 weeks of irradiation. The incidence of tumor-bearing animals among those still alive after 65 weeks reached 75% in the MnPV^+^ UV-irradiated colony, whereas it did not exceed 18% in MnPV^-^ animals. Notably, eight MnPV^+^ animals (all within the 450 mJ/cm^2^ group) additionally developed tumors at unirradiated sites (in the ear, around eyes or mouth), an observation that may be attributed to a systemic immunosuppressive effect mediated by chronic UVB exposure [36].

These results show that UVB irradiation and cutaneous PV infection strongly promoted tumor formation in MnPV^+^ animals that developed lesions significantly more frequently than MnPV^-^ animals (p = 0.0009, Mantel-Cox test) or their unirradiated counterparts (p<0.0001). Although the cumulative UVB dose was different for the three dose groups (Fig 1A) no significant dose-response relationship could be noted (S1 Fig).

### UV-induced skin tumors comprise two distinct types of squamous cell carcinomas

In the course of a natural infection, MnPV can induce the development of benign skin lesions such as papillomas and keratoacanthomas (Fig 1C) [15,21]. Monitoring UV-irradiated MnPV^+^ animals, however, two entities of skin tumors were observed. The first developed to larger keratinized nodules, which often contained a central keratin plug surrounded by atrophic skin (Fig 1D). These keratinizing SCCs (KSCC) morphologically and histologically resembled keratoacanthomas in humans [37,38] and were macroscopically indistinguishable from spontaneous tumors in naturally infected animals. The presence of koilocytes in these lesions (S2 Fig, right panels) suggests productive PV infections [39]. Conversely, the second tumor phenotype, referred to as non-keratinizing SCCs (nKSCC), grew faster (Fig 1E). These relatively flat but deeply infiltrating tumors often developed ulcerations as they became larger. Notably, in the beginning some tumors had macroscopic similarities with KSCCs and later partially developed to an nKSCC (S3 Fig). Moreover, nKSCCs were found to be more prominent in the groups with higher UV doses, suggesting that increased irradiation damage influenced the tumor type (Fig 1F).

**Fig 2:**
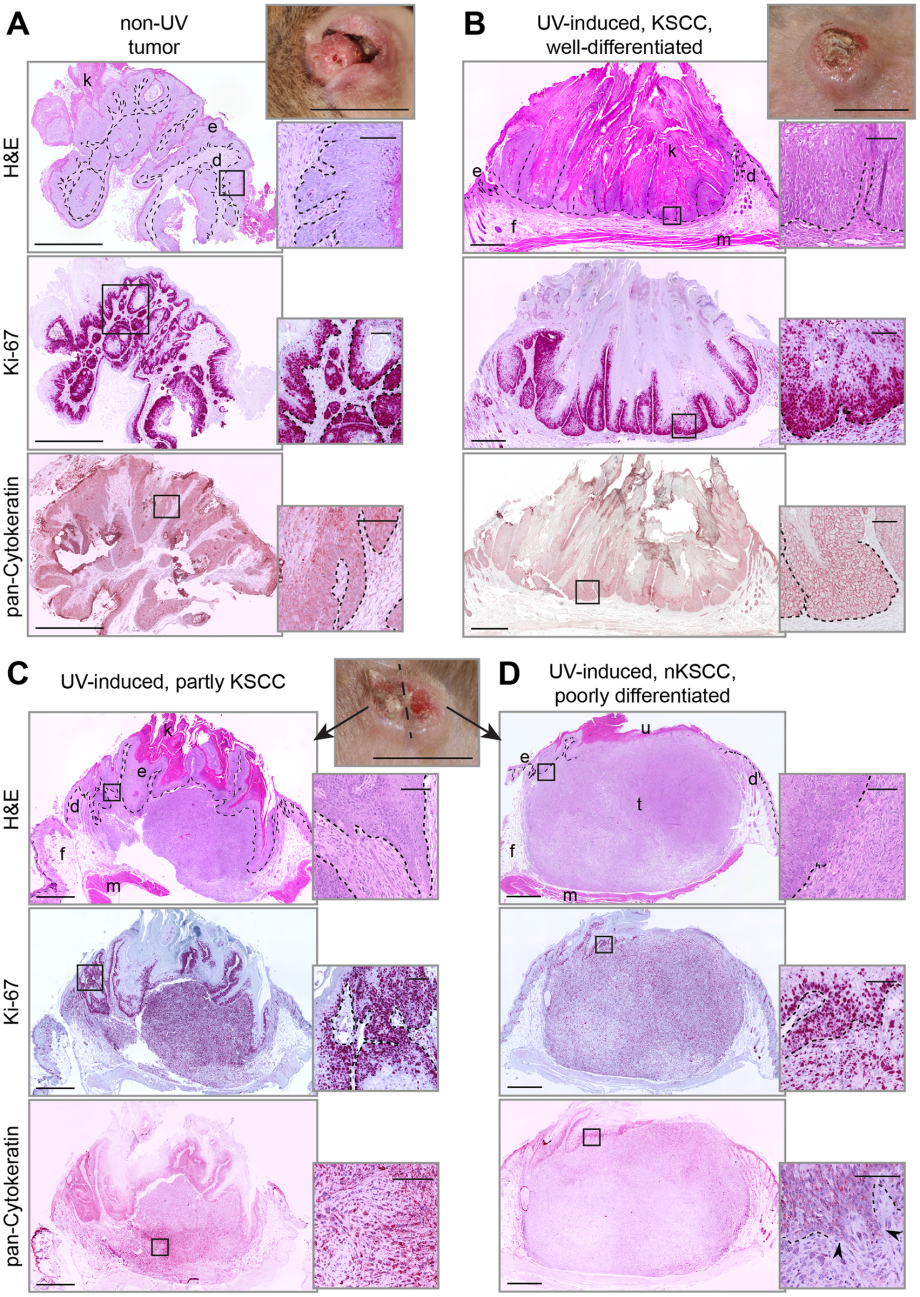
Histological analyses of a non-UV tumor and UV-induced tumors. **A)** Tumors from unirradiated sites show papilloma-like growth of well-differentiated neoplastic squamous cells (H&E). Especially the basal layers are hyperproliferative as indicated by strong Ki-67 staining. Throughout all layers of the lesion, neoplastic cells strongly express cytokeratins (pan-Cytokeratin). **B)** A UV-induced KSCC with well-differentiated exoendophytic proliferations of squamous cells expressing Ki-67 throughout all neoplastic squamous layers. **C and D)** In some cases well-differentiated KSCCs **(C)** further developed into more aggressive poorly differentiated nKSCCs **(D)**. Proliferating altered squamous cells thereby invaded deeper layers and often changed to a spindle-like phenotype (H&E). The Ki-67 staining becomes diffuse in this process (compare insets) and cytokeratin expression is reduced. (d: dermis; e: epidermis; f: fat; k: keratin; m: muscle; u: ulceration; t: tumor. Scale bars: macroscopic: 10 mm, overviews: 1 mm, insets 100 μm).

**Fig 3:**
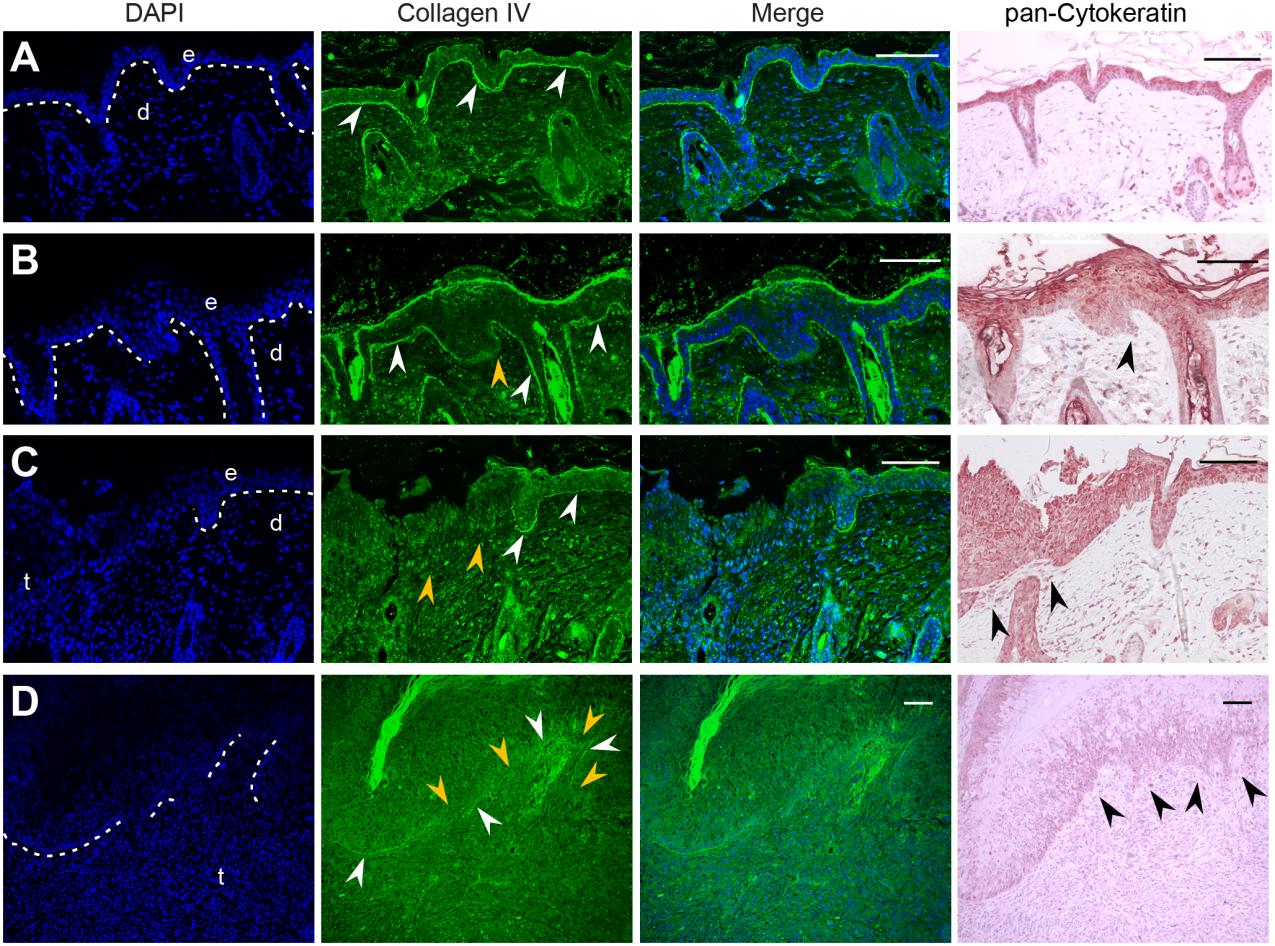
Collagen IV staining on tissue sections reveals invasion of keratinocytes through the basal membrane (BM). The BM was stained against collagen IV (green). Nuclei were counterstained with DAPI (blue). Consecutive sections stained for pan-cytokeratin are shown in comparison. **A)** In normal skin, the BM (white arrows) marks the barrier between epidermis and dermis. **B)** Early stage carcinoma formation in UV-irradiated skin. A lack of collagen IV expression indicates the disruption of the BM (orange arrows) accompanied by downward migrating cells (black arrow). **C)** In the edge region of a UV-induced KSCC, the BM is lost and invading altered keratinocytes are detectable. **D)** In nKSCC, invasion of neoplastic cells is advanced as indicated by pan-cytokeratin staining. The discontinuous staining of the BM marks transition zones where invading neoplastic squamous cells acquire a spindle cell phenotype (Scale bars: 100 μm).

Histologically, and similarly to tumors from unirradiated sites (Fig 2A), KSCCs were characterized as exoendophytic multilocular proliferations of well-differentiated neoplastic squamous epithelium with different degrees of parakeratosis (Fig 2B). Hyperproliferative Ki-67-positive cell layers were broadened and atypical keratinocytes showed expression of keratins. In a transitional tumor, combining features of KSCCs and nKSCCs (Fig 2C and 2D), areas of well-differentiated, hyperproliferative, atypical squamous cells converted into pleomorphic cells. These often showed a spindle cell phenotype and a diffuse Ki-67 staining. However, although still expressing cytokeratins, they changed their phenotype when migrating out to invade deeper layers (Fig 2D, see arrows in inset), thereby forming a less differentiated tumor. Furthermore, immunofluorescence for the basal membrane major component collagen IV displayed a continuous staining of the dermo-epidermal junction in normal skin, which was disrupted in an early stage carcinoma after UV-irradiation, in KSCCs as well as in nKSCCs (Fig 3).

### High MnPV DNA loads and transcription in well-differentiated KSCCs but not in poorly differentiated nKSCCs

**Fig 4:**
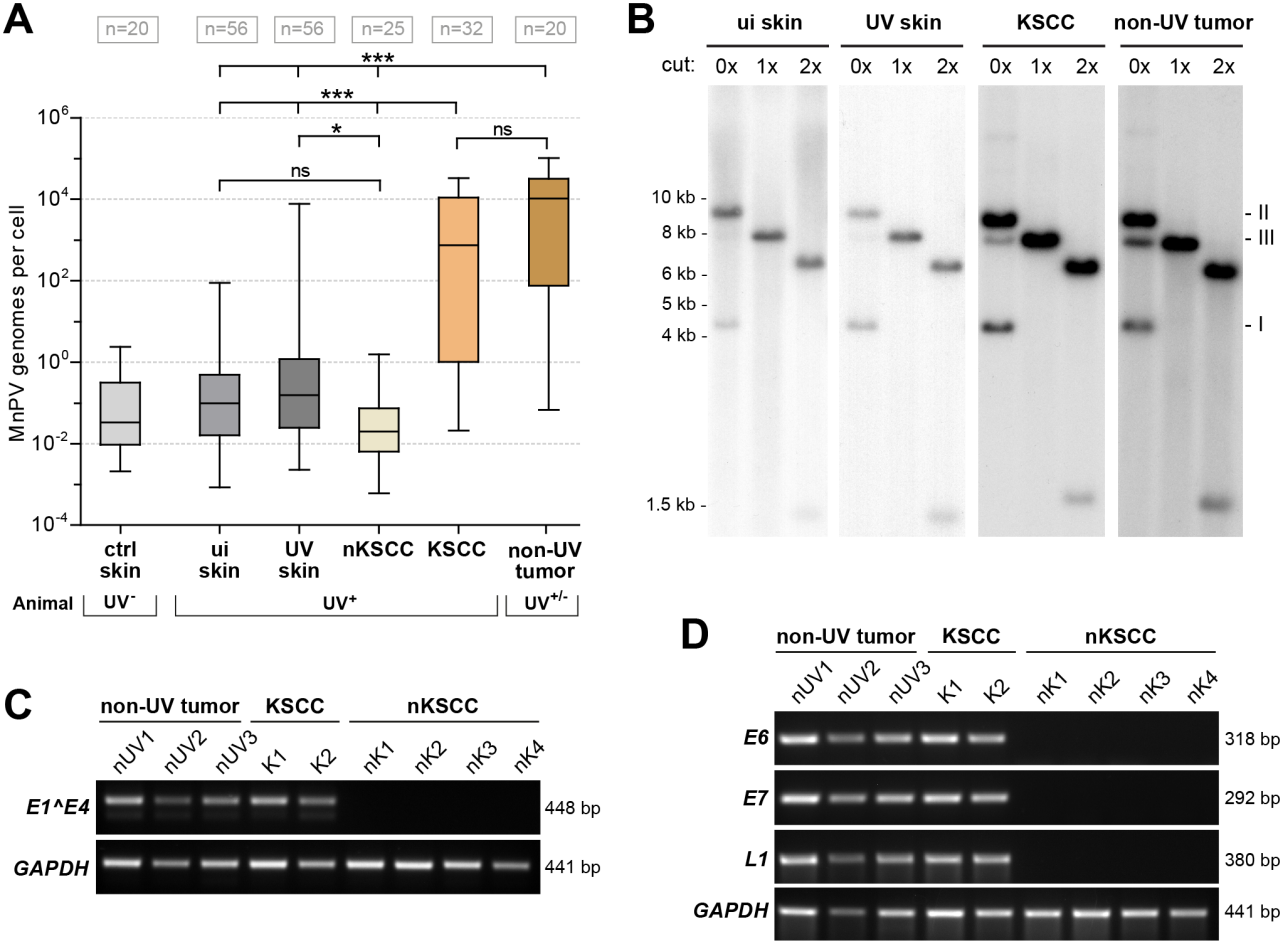
Molecular analyses of tumor-bearing animals. **A)** Viral load in tissue samples from UV-irradiated and control animals from the MnPV-infected colony analyzed by qPCR and normalized to a plasmid standard. Samples were grouped according to their origin as indicated (ctrl skin: skin from unirradiated animals; ui skin/UV skin: unirradiated or UV-irradiated skin from irradiated animals; KSCC/nKSCC: UV-induced SCCs; non-UV tumor: tumors from non-UV sites of irradiated animals and spontaneous tumors from unirradiated animals). UV^+/-^ indicates whether the animal was UV-exposed or not (Kruskal-Wallis test, *p<0.05, ***p<0.001, ^ns^p>0.05). **B)** Southern blot analysis of unirradiated and UV-irradiated skins, a KSCC and a non-UV tumor. DNA was digested with ApaI (no cleavage site in MnPV), XbaI (one site) or XhoI (two sites) as indicated (Form I: supercoiled; Form II: relaxed circular; Form III: linear form of MnPV). **C)** Semi-quantitative RT-PCR for the most abundant MnPV *E1^E4* transcript in non-UV tumors and UV-induced SCCs or the control *GAPDH*. **D)** Semi-quantitative RT-PCR for MnPV *E6*, *E7* and *L1* transcripts in non-UV tumors and UV-induced SCCs or the control *GAPDH*.

Human NMSCs either completely lack cutaneous HPVs or contain very low DNA loads [10], indicating that viral oncoproteins are apparently not necessary to maintain a proliferative and tumorigenic phenotype [40]. To determine whether the SCCs, representing distinct differentiation states, contain different viral loads, we measured the amount of MnPV DNA in UV-induced tumors by quantitative PCR. As shown in Fig 4A, no significant differences in viral copy numbers were found in unirradiated and irradiated skins (S1 Table). Of note, however, UV-induced SCCs significantly differed in their viral load. Well-differentiated KSCCs had a significantly higher viral load compared to normal skin, reaching values also observed in MnPV-induced papillomas and keratoacanthomas where virus production is taking place [17]. Southern blot analyses of DNA obtained from different lesions and normal skin showed episomal supercoiled and nicked circular DNA without any indication for integration (Fig 4B). In contrast, the viral load in nKSCCs was significantly lower than in KSCCs and comparable to unirradiated skin (Fig 4A).

**Fig 5:**
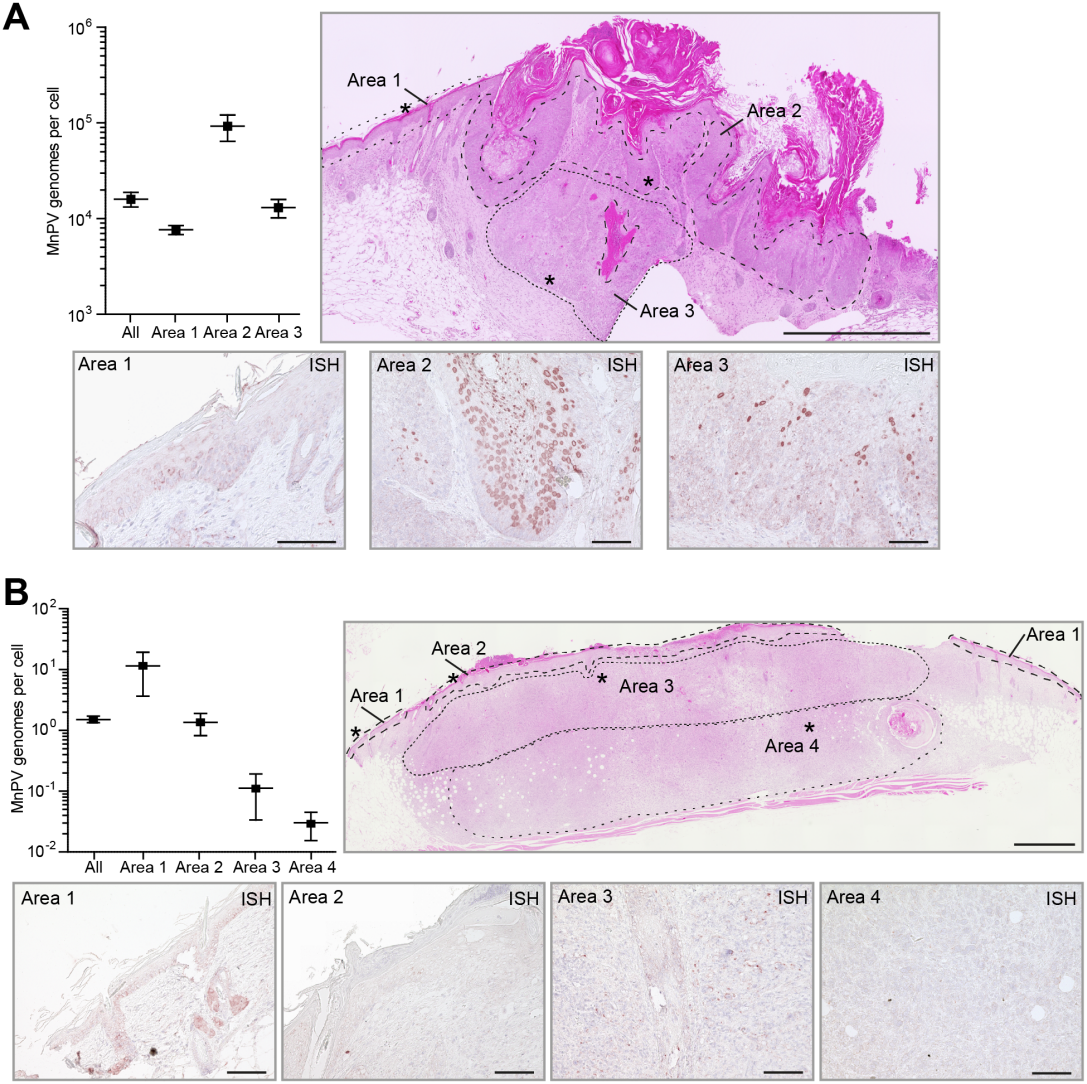
Spatial analysis of viral load in UV-induced SCCs. Quantification and distribution of MnPV DNA in different microdissected areas (specified in the HE staining) of **A)** a KSCC and **B)** a UV-induced nKSCC. Asterisks indicate the position of the respective MnPV-specific *in situ* hybridization (ISH) (Scale bars: HE: 1 mm, ISH: 100 μm).

To determine whether MnPV is transcriptionally active in these tumor entities, semi-quantitative RT-PCRs for the spliced *E1^E4* transcript were performed. Consistent with other papillomaviruses [41], this was also the most abundant MnPV transcript in productive skin lesions of *Mastomys coucha* [42]. The corresponding mRNAs could only be detected in tumors from unirradiated sites and KSCCs that contained high viral loads, but not in nKSCCs with low copy numbers or MnPV-negative lesions (Fig 4C) (see viral loads in S2 Table). However, to examine the activity of the early and late promoter in these lesions, we further analyzed tissue samples for E6/E7 and L1 transcription. As shown in Fig 4D, while still expressed in KSCCs, none of these transcripts could be detected in nKSCCs. These results indicate that tumors with an nKSCC phenotype counter-select for permissive MnPV production due to dedifferentiation that may explain the quantitative loss of viral copies during malignant progression. To further substantiate this assumption, we microdissected different areas from KSCCs and nKSCCs in order to match the degree of differentiation with the spatial distribution of viral loads. As depicted in Fig 5A, KSCCs indeed showed a heterogeneous staining pattern for the viral DNA when different areas were examined by *in situ* hybridization (ISH). Variations in the copy number could also be confirmed by real-time qPCR after extracting DNA obtained from corresponding tumor regions. In contrast, monitoring nKSCCs, viral copy numbers decreased from upper to lower dedifferentiated layers, being below the detection limit in the ISH (Fig 5B). Here, based on real-time qPCR analyses, only every 10^th^ or 100^th^ cell retained viral DNA. Concomitantly, IHC against Ki-67 revealed strong proliferation throughout basal and suprabasal layers in virus-positive KSCCs, while only a dispersed pattern could be discerned when virus-negative nKSCCs were examined (S4 Fig, see also Fig 2).

### UV-irradiated animals develop antibody responses against MnPV capsids

**Fig 6:**
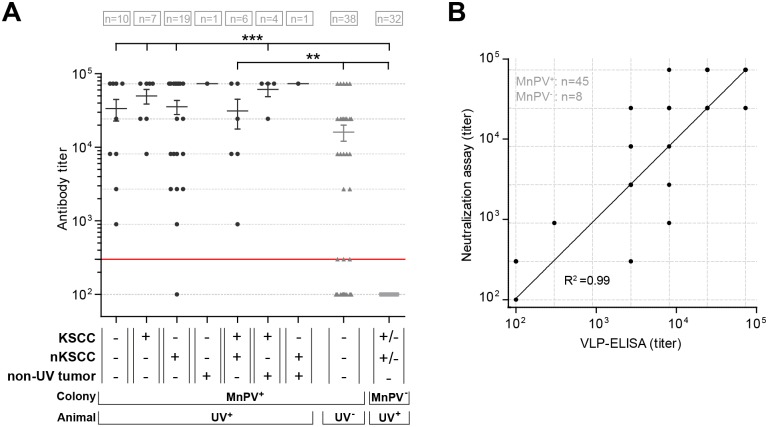
Serological analyses of the animals. **A)** Antibody responses of UV-irradiated and control animals against MnPV-L1-VLPs. Final sera of 55 to 75 week old animals were measured. Animals were grouped according to their origin (MnPV^+^ or MnPV^-^ colonies) and treatment. Different groups represent distinct constellations of tumor types in the animals. Note that both MnPV^-^ tumor-bearing animals were included in the last group. The cut-off for the assay is indicated by the red line (titer of 300) (Mean ± SEM; Kruskal-Wallis test, **p<0.01, ***p<0.001). **B)** Correlation of pseudovirion-based neutralization titers and antibody titers measured by VLP-ELISA. The non-linear fitting indicates a correlation of 99% between both assays.

While quantitative differences in the viral load only reflect the situation at a defined time point, seroresponses against viral capsids represent a more reliable marker of preceding infections. Indeed, antibody titers against cutaneous HPV types are very stable in NMSC patients [14,43]. To recapitulate an infectious history, we monitored final sera of all our animals for seroresponses against MnPV virions in a VLP-ELISA [21]. All animals with UV-induced tumors, except for one case with an nKSCC, showed high titers of MnPV-specific antibodies which was not the case to such an extent for MnPV^+^ unirradiated animals (Fig 6A). Tumor formation could not be attributed to an inefficient humoral immune response, since these antibodies had neutralizing capacity when pseudovirion-based infection assays were applied [44] (Fig 6B). Animals in the MnPV^-^ colony displayed no seroresponses against MnPV.

### Induction of γH2AX foci and diminished CPD repair in MnPV-positive cells and tissue sections

**Fig 7:**
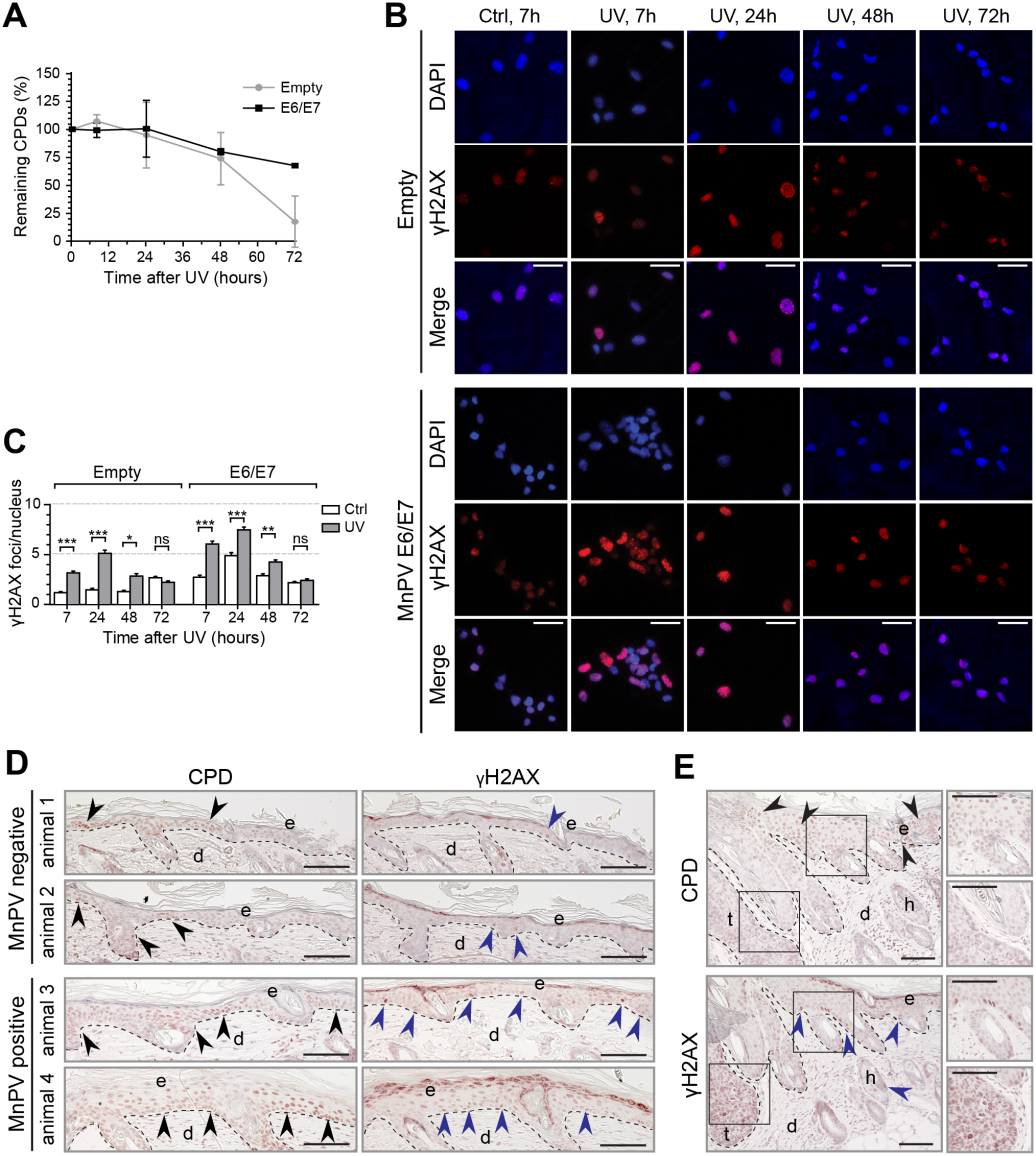
MnPV interferes with DNA damage repair. **A)** Repair kinetics of CPDs in MnPV E6/E7-positive and -negative *Mastomys* keratinocytes (Mean ± SD; n = 2, measurements were performed in quadruplicates). **B)** Immunofluorescence staining of γH2AX foci in keratinocytes stably expressing MnPV E6/E7. Cells were irradiated with UVB and further incubated prior to detection and quantification of γH2AX foci (Ctrl: unirradiated, UV: irradiated; Red: γH2AX, blue: nuclei; scale bars: 50 μm). **C)** Quantification of γH2AX foci (Mean ± SEM; n≥242; 1way-ANOVA, *p<0.05, **p<0.01, ***p<0.001). **D)** Co-detection of CPDs and γH2AX in MnPV^+/-^ skin harvested 24h after UV irradiation. Arrows point towards positive cells (Viral loads: animal 3: 13.68 ± 1.66 copies/cell, animal 4: 147.42 ± 14.62 copies/cell; Scale bars: 100 μm). **E)** Co-detection of CPDs and γH2AX in a KSCC harvested 24h after UV irradiation (Viral load: 611.88 ± 18.75 copies/cell; scale bars: 100 μm).

Beta-HPVs developed strategies to interfere with the repair machinery of their host cell which may have deleterious effects, particularly when UV exposure is involved [45–50]. To monitor these issues in our experimental system, we first analyzed the CPD repair *in vitro*. Here, *Mastomys* coucha keratinocytes [51] expressing retrovirally transduced MnPV E6/E7 and virus-negative controls were irradiated with UV and incubated for different periods of time. As demonstrated in Fig 7A, MnPV E6/E7 attenuates CPD repair 72h after UV exposure, indicating an impact of MnPV on the DNA damage response. Since persisting CPDs can lead to an accumulation of phosphorylated H2AX (γH2AX) [52], a surrogate marker for DNA damage and chromosomal instability [53,54], we further examined the impact of MnPV E6/E7 expression and UVB on γH2AX foci formation by immunofluorescence (Fig 7B). While there was also a weak response in virus-negative cells as a consequence of UV exposure, in E6/E7-expressing keratinocytes more foci could be quantified after 7h and 24h (Fig 7C). In control cells, these disappeared faster than in cells expressing E6/E7, which *per se* showed a higher amount of γH2AX foci even without prior irradiation. Notably, a similar effect could also be observed *in vivo* by comparing representative skin sections obtained from two MnPV^-^ and MnPV^+^ animals that were sacrificed 24h after the last irradiation. Here, epidermal keratinocytes of uninfected animals showed only a sporadic staining for CPDs and γH2AX, while skin sections of MnPV^+^ *Mastomys* exhibited a stronger positivity for both markers (Fig 7D). The skin surrounding a KSCC shown in Fig 7E also harbored keratinocytes positive for both CPD and γH2AX, while deeper layers of the KSCC itself were strongly positive for γH2AX but negative for CPDs (see insets).

**Fig 8:**
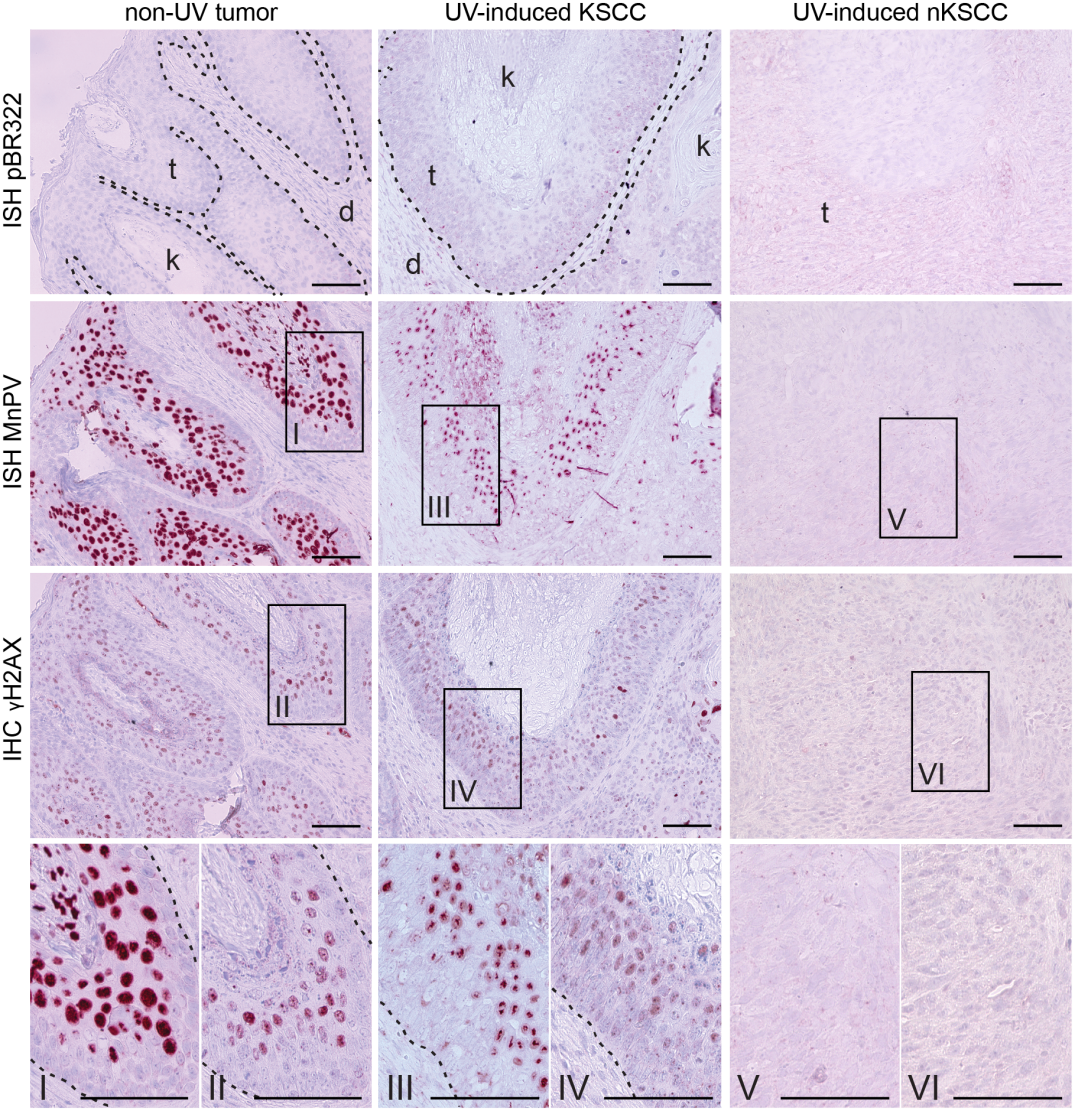
Induction of γH2AX foci by MnPV. Co-detection of γH2AX and MnPV DNA in consecutive tissue sections. Left panel: γH2AX staining of a non-UV tumor correlates with high viral load detected by ISH with a MnPV-specific probe (Viral load: 12065.07 ± 1119.24 copies/cell). Middle panel: the same concurrence can be detected in UV-induced KSCCs (Viral load: 26592.94 ± 1823.92 copies/cell). Right panel: UV-induced nKSCCs are negative in both stainings (Viral load: 0.98 ± 0.1 copies/cell; scale bars: 100 μm).

To determine the impact on DNA damage response in correlation with the viral load, tumor sections were stained with an antibody directed against γH2AX. In parallel, consecutive sections were examined by ISH for the presence of MnPV. Here, consistent with the quantification of the viral loads (Fig 4A), a strong staining for MnPV DNA could be visualized in suprabasal layers of tumors from unirradiated sites and in UV-induced KSCCs (Fig 8). Intriguingly, there was a clear coincidence of ISH and γH2AX signals in both lesions, indicating that apparently the mere presence of MnPV already activates a kind of DNA damage response in terms of γH2AX foci formation, even in a tumor never exposed to UVB. In contrast, nKSCCs with low or lacking viral loads were negative for γH2AX. Since γH2AX foci only exist temporarily and disappear after DNA damage repair [55], their persistence in non-UV tumors and KSCCs indicates a continuous interference with the host genome in MnPV-infected cells that may also contribute to maintain their replication levels in a still permissive environment.

### MnPV E6 does not affect p53 transactivation efficiency

**Fig 9:**
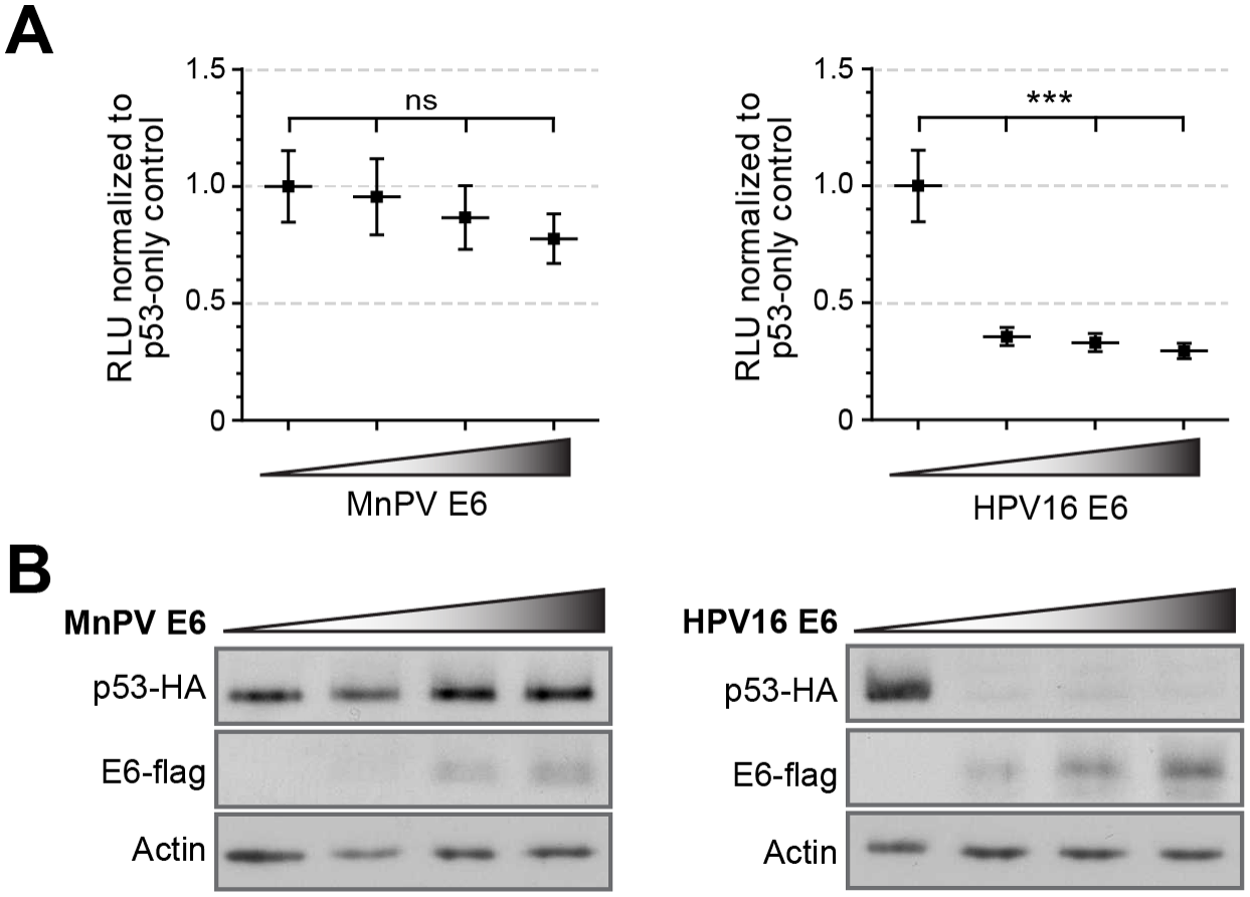
Transactivating capacity of *Mastomys* p53 in the presence of MnPV E6. **A)** The capacity of p53 to transactivate a p53-responsive firefly luciferase gene measured in H1299 cells transfected with reporter plasmids and expression vectors for *Mastomys* p53 and MnPV E6 or human p53 and HPV16 E6 as a control. Transactivation activity was measured by luminescence (RLU, relative light units). Cells transfected only with p53 served as control and their RLU levels were arbitrarily set to 1 (Mean ± SEM; n = 7; 1way-ANOVA, ***p<0.0001). **B)** Western blots showing protein levels of p53 and E6 in the lysates of the transactivation assay. Actin served as an internal loading control.

Although most of the cutaneous HPV types cannot degrade p53, they can impede intracellular signal transduction of p53, p53 family members, downstream pro-apoptotic proteins and proteins involved in DNA repair [26–29,56]. To investigate the impact of MnPV E6 on p53 in the context of tumor formation, we co-transfected cloned *Mastomys coucha* p53 together with MnPV-E6 and tested its effect in p53-luciferase reporter assays (Fig 9A). Western blot analyses were used to monitor quantitative changes of both proteins in comparison to actin (Fig 9B). While the transactivating activity of human p53 was completely abrogated by HPV16 E6 due to degradation (Fig 9A, right panel), there was only a marginal effect on reporter activity when increasing amounts of MnPV E6 were transfected (Fig 9A, left panel), despite unaffected steady-state levels of p53 (Fig 9B).

### UV-induced skin tumors in *Mastomys coucha* acquire mutations in *Trp53* similar to human SCCs

**Fig 10:**
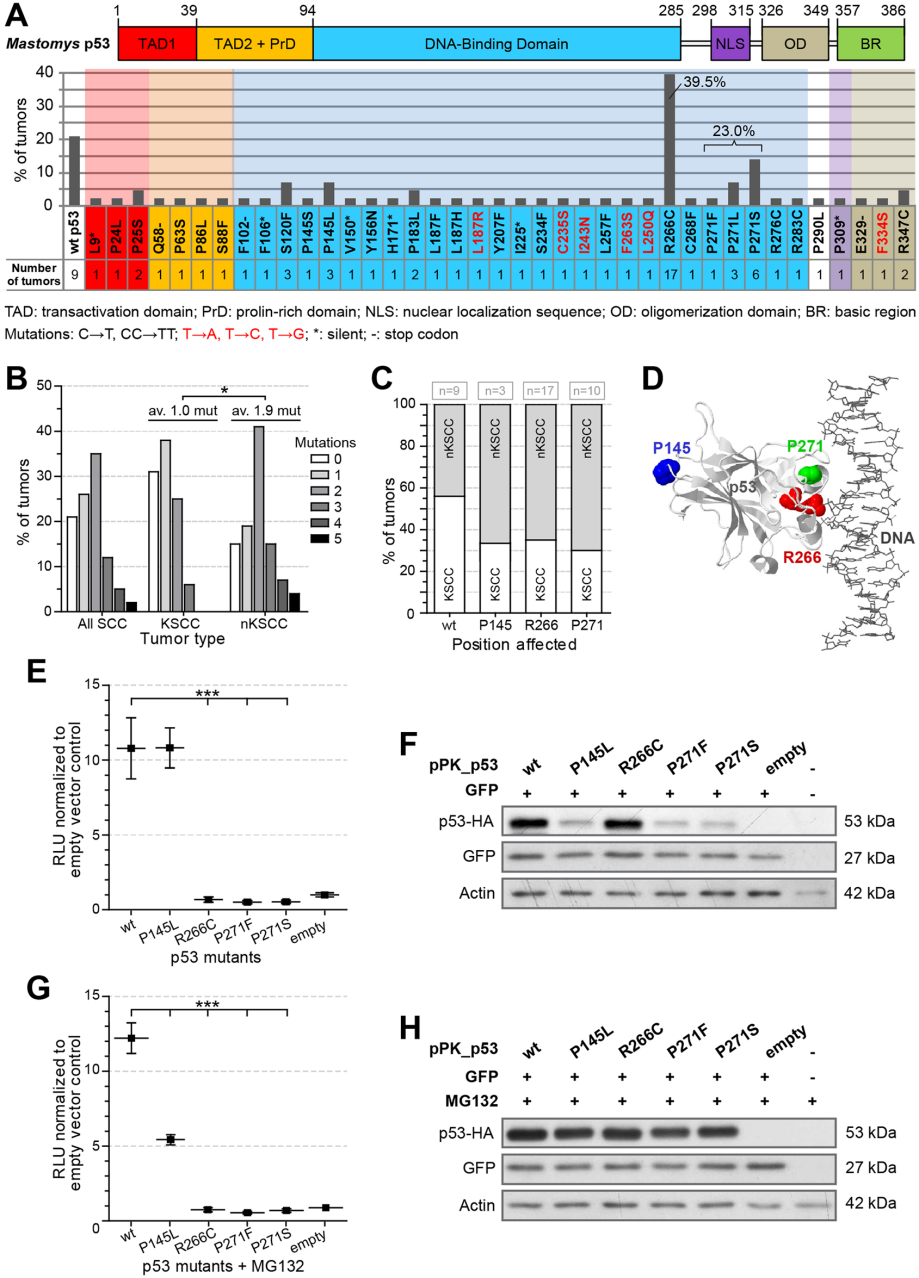
Analysis of the *Trp53* status in UV-induced SCCs. **A)** Schematic structure of *Mastomys coucha* p53 (based on [113]) matched to the position and the frequency of mutated residues (n = 43). Note that residue P271 was substituted by three different amino acids. **B)** Comparison of the number of *Trp53* mutations in KSCCs (n = 16), nKSCCs (n = 27) and the total number of SCCs (n = 43) (unpaired t-test, p = 0.0227). **C)** Frequency of mutations at positions P145, R266 and P271 in KSCCs in comparison to nKSCCs. **D)** Locations of the hot-spot mutations in a 3D model of p53. Selected residues are highlighted (source: http://p53.iarc.fr/MakeJMol.aspx). **E)** Hot-spot mutants were cloned in an expression vector and tested for their capacity to transactivate a p53-responsive firefly luciferase gene. Transactivation activity was measured by luminescence (RLU, relative light units). Cells transfected with empty vector served as control and their RLU levels were arbitrarily set to 1 (Mean ± SEM; n = 7; 1way-ANOVA, ***p<0.001). **F)** Western blot showing protein levels of p53 mutants measured in the transactivation assay. EGFP was used as a control for transfection efficiency, actin as an internal loading control. **G)** Same as shown in panel E. Prior to the measurement of the transactivation, transfected cells were treated with 5 μM MG132 (Mean ± SEM; n = 4; 1way-ANOVA, ***p<0.001). **H)** Western blot of transfected cells after treatment with 5 μM MG132.

Since p53 is a major decisive factor in sensing DNA damage and due to its high mutation frequency in human cancer [57,58], we sequenced *Trp53* cDNA from UV-induced tumors and analyzed its functional status. Human and *Mastomys coucha* p53, especially the DNA-binding and the oligomerization domains, are extremely conserved, anticipating that mutations within these regions may have the same functional impact as found in humans [59]. In 79% of UV-induced SCCs (34 out of 43 samples) at least one mutation was present, which was not the case for UV-irradiated skins (n = 7), unirradiated skins (n = 4) and lesions from unirradiated sites (n = 4), respectively. As expected, most mutations were UV-induced C→T and CC→TT transitions. Fig 10A matches frequency and localization of the mutations to the predicted domain structure of *Mastomys coucha* p53. Similar to human skin cancer [57], residues R266 and P271 (R273 and P278 in human p53) in the C-terminal part of the DNA-binding domain were found to be hot-spots for UV-induced mutagenesis. Allocating *Trp53* mutations in the context of their histopathological origin, nKSCCs harbored significantly more mutations than KSCCs (Fig 10B). Whether these cells harbor multiple *Trp53* mutations or whether this reflects tumor heterogeneity [60] remains to be elucidated. Nonetheless, since mutations at hot-spots R266 and P271 were mostly found in nKSCCs, they apparently favored the development of a more aggressive phenotype and seem to be inversely correlated to the viral load (Fig 10C). An *in silico* modelling of the binding of *Trp53* to DNA (using human p53 with correlating positions) clearly indicates the importance of R266 and P271 which are either directly involved in the DNA binding or at least located in close proximity (Fig 10D).

To analyze the mutational impact on *Mastomys coucha* p53, we ectopically expressed hot-spot mutants R266C, P271F and P271S, as well as mutant P145L in H1299 cells and tested their ability to transactivate a p53-responsive reporter (Fig 10E). Here, the hot-spot mutants completely lacked transactivation activity, whereas the more distal P145L mutant, which was detected with less frequency in tumors, showed the same activity as wildtype p53. Furthermore, P145L, P271F and P271S were also less stable (Fig 10F). Conversely, although R266C was not affected in its intracellular half-life, its ability to transactivate was lost. Even the addition of the proteasome inhibitor MG132 could not increase the transactivation efficiency of hot-spot mutants P271F and P271S (Fig 10G), although their protein levels were stabilized (Fig 10H). In contrast to wildtype p53, the activity of P145L decreased when MG132 was applied, probably due to cofactor squelching which counteracts its functionality [61].

### IHC staining of mutant p53 in nKSCCs

Since elevated levels or stable forms of mutated p53 are frequently found in cancer cells [58], we also stained tissue sections of UV-irradiated MnPV^+^ animals for p53 and cytokeratin expression. While undetectable in unirradiated skin, p53-positive islands of squamous cells were visible in UV-irradiated skin (S5A and S5B Fig). Furthermore, atypical squamous cells that migrated out of the hyperproliferative epidermis of nKSCCs acquired a spindle-like morphology and enhanced p53 levels (S5C and S5D Fig).

**Fig 11:**
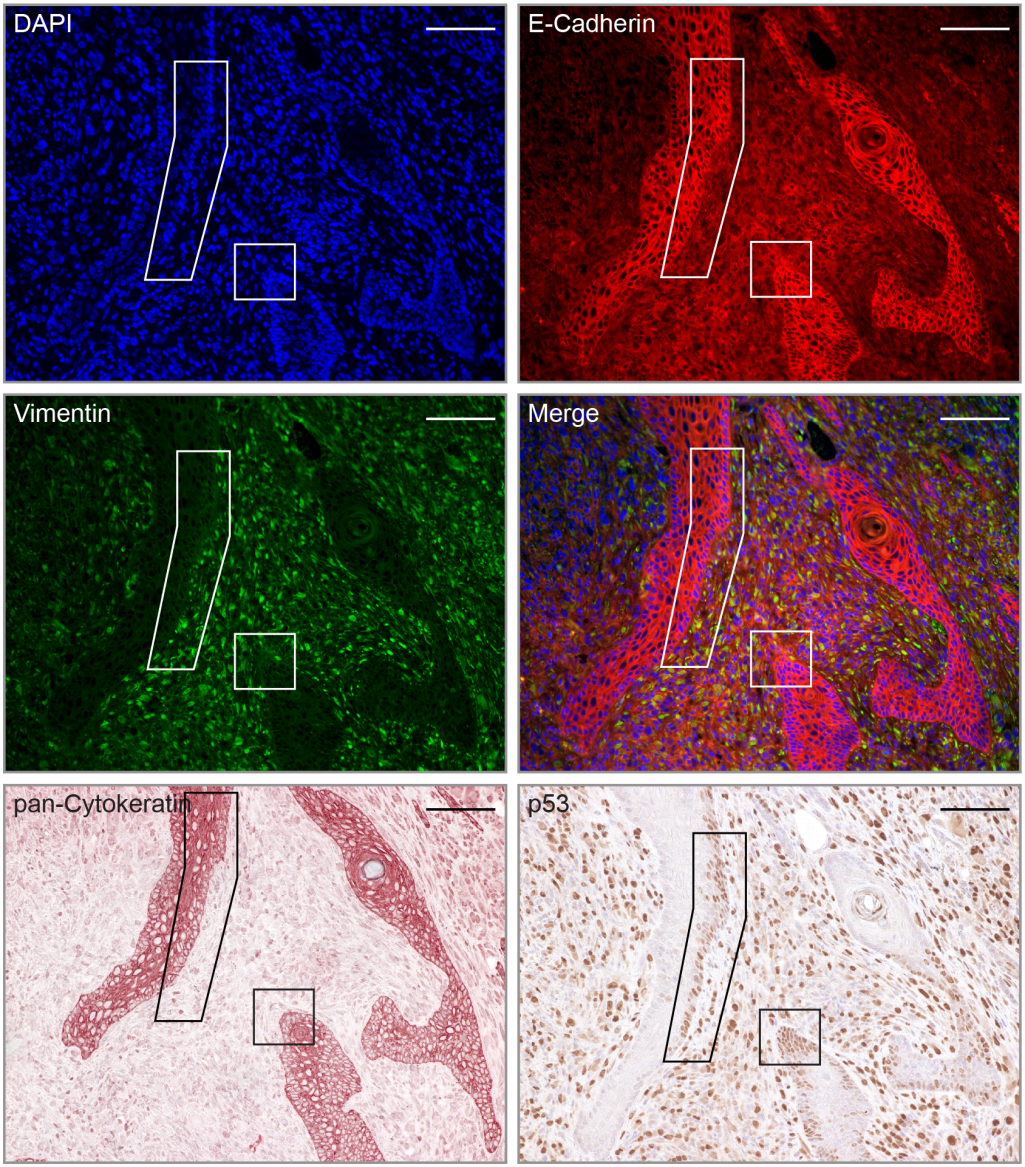
Dedifferentiation correlates with positive p53 staining. Consecutive sections of a poorly differentiated nKSCC were stained with antibodies against E-cadherin, vimentin, pan-Cytokeratin and p53. DAPI was used as nuclear counter stain. Note that in this tumor, only mutation R266C could be detected (Scale bars: 100 μm).

As reported elsewhere, *Trp53* knockout leads to spindle cell SCCs in mice [62], suggesting dedifferentiation after loss of functional p53 [63]. To examine this possibility for nKSCCs, we stained consecutive tumor sections where reduced pan-cytokeratin and E-cadherin levels matched with increased intensity of vimentin (Fig 11). Furthermore, these stainings strongly coincided with p53-positive areas (see frames in Fig 11) in zones where transition of differentiated cells into undifferentiated cells takes place. Since R266C was the only *Trp53* mutation found in this nKSCC, we argue that there is a strong relationship between loss-of-function *Trp53* mutations and dedifferentiation [64,65]. This may favor the development of tumors independently from viral oncogene expression.

## Discussion

Considering that more than 95% of all viral sequences found in human skin belong to β- and γ-HPVs [66], it is essential to investigate whether a functional interaction between UV and HPV infection exists. Indeed, both molecular and epidemiological data support a functional contribution of some cutaneous HPVs in the time-dependent multistep process of NMSC development [67,68]. However, there is still incongruity since viral DNA is lost during progression from actinic keratosis to cutaneous SCC [69]. This contrasts with cervical cancer where integrated high-risk α-HPV DNA is detectable in all tumor cells [70].

Although well studied in transgenic mice [71], research on the role of cutaneous PVs in skin carcinogenesis is still hampered by the lack of suitable naturally infected animal models that recapitulate a complete productive viral life cycle and subsequent immune challenge to reflect the situation in patients. The MmuPV1 mouse model readily allows efficient formation of cutaneous lesions. However, the amounts of viral particles needed for an experimental infection highly exceed those causing natural infections and is only successful at defined anatomical sites and in certain mouse strains [72,73].

Here, we used the naturally MnPV-infected and immunocompetent rodent *Mastomys coucha* to show for the first time a functional link between cutaneous PV infection, UVB irradiation and NMSC development. Our results differ from previously published models. While in transgenic HPV8-E6 mice [30,33] and experimentally MmuPV1-infected mice [73], a single UVB dose was sufficient for SCC formation, more than 30 weeks of continuous treatment were necessary until the first lesions appeared at the backs of our animals (Fig 1D and 1E). Hence, these kinetics resembles more the time course of tumor development found in HPV38-E6/E7 mice [32] for which a similar irradiation protocol was used (Fig 1B). The time range also better reflects the development of cutaneous SCCs in humans, which are linked to cumulative life-long sun exposure and appear mostly in elderly patients [74,75]. Moreover, based on the contact hypersensitivity reaction of the skin [76], a broad range of UV susceptibility in diverse rodent strains could be noted and even hair pigmentation seems not to account for these differences since C57 mice (highly pigmented) are much more susceptible to UV than BALB/c albino mice ([77] and references herein). We therefore applied three different final doses of UVB to monitor the onset of tumor development, but the kinetics of NMSC development was independent of the intensities used here (S1 Fig). It became evident, however, that MnPV^+^ *Mastomys coucha* developed skin tumors at the UVB-irradiated back significantly more frequently than MnPV^-^ irradiated or MnPV^+^ unirradiated controls, clearly demonstrating a cooperative effect between MnPV infection and UV on SCC formation (Fig 1B).

UVB intensity, however, influenced the appearing tumor type. The cohort irradiated with a final dose of 450 mJ/cm^2^ UVB more often developed KSCCs (Fig 1D and 1F) compared to animals that received higher doses, in which especially nKSCCs were obtained (Fig 1E and 1F). The location of SCCs in *Mastomys coucha* also contrasts with the MmuPV1 mouse system where lesions preferentially develop at the tail, muzzle or ear, while the back skin was less predisposed or even resistant [19,72]. The putative underlying reason was recently addressed in an elegant study using quantitative trait loci network analysis in mice [78]. According to this report, dorsal and tail skin is not only dissimilar by different keratin networks and transcription factors, but also due to their lower expression of markers for tissue-resident Langerhans cells and MHC expression. Since both are involved in the immune response against PVs [79], their depletion may account for the local susceptibility to MmuPV1 infection and papilloma formation in the murine system.

The tumor entities found in our study could be histopathologically identified either as a well-differentiated type (KSCC, Fig 2B) or as a more aggressively growing poorly differentiated type (nKSCC; Fig 2D) [38], mainly composed of deeply invading pleomorphic spindle cells infiltrating the underlying dermis (Fig 3). Human cutaneous SCCs with a spindle cell component could be found in almost 20% of immunosuppressed patients, showed aggressive growth [80] and usually appear in heavily sun-damaged skin areas [81]. It is therefore tempting to speculate that nKSCCs preferentially obtained at higher UVB doses represent a more progressed phenotype as a result of the acquisition of additional driver mutations during carcinogenesis. This is in line with the observation of some intermediate tumors evolving from KSCCs (S3 Fig). While previous studies have not yet shown a correlation between viral load and the differentiation status of SCCs, we addressed this question in our preclinical model.

Regarding MnPV DNA, KSCCs harbored viral loads comparable to tumors appearing at sites unexposed to UVB, although a trend towards lower amounts could be noted (Fig 4A). In both entities, MnPV was episomal (Fig 4B) and transcriptionally active as demonstrated by detection of the spliced *E1^E4* transcript, previously found to be the most abundant in productive infections [42]. These lesions still showed early and late transcription, since E6/E7 and L1 specific transcripts could be detected (Fig 4C and 4D). Conversely, the viral load was significantly lower or even absent in nKSCCs (Fig 4A, see also S1 Table) that in turn of course also explains the lack of viral mRNA in these tumors (Fig 4C and 4D). This is consistent with epidemiological studies reporting still high levels of transcriptionally active HPV in actinic keratoses, but not in SCCs [69]. The significant loss of viral DNA in nKSCCs also diverges from another model hitherto used for skin carcinogenesis, namely the infection of domestic rabbits with cottontail rabbit PV (CRPV) [82]. Here, malignant skin tumors still contain high copy numbers of transcriptionally active CRPV DNA [83], thereby only limitedly reflecting the situation in humans.

The viral load only represents a current status that may change during the multi-step process of skin carcinogenesis [5]. Therefore, a more reliable parameter for detecting a preceding infection—especially in animals that developed nKSCCs with negligible residual amounts of viral DNA in these tumors—was the determination of antibody responses against MnPV capsids using a VLP-ELISA (Fig 6A) [21]. Seroconversion is very stable, especially for cutaneous HPVs [84], and therefore represents the only proof of an infectious history in SCCs when viral DNA is barely discernible or absent [43]. Newborn and still uninfected *Mastomys coucha* are completely seronegative for MnPV early and late proteins, but develop strong responses several weeks after viral infection [21]. Although serum responses showed considerable individual variability, animals with well-differentiated KSCCs had the highest titers which probably can be attributed to a stronger immune exposure due to a productive viral infection. The titers were more dispersed in the group of animals with poorly differentiated nKSCCs than in those with KSCCs (variation coefficient 94% vs. 59%, p<0.001), which may reflect the quantitative loss of MnPV in these lesions (Figs 4A and 5) and the absence of a continuous immune challenge. Thus, dedifferentiation of squamous cells interferes with viral replication and maturation [85], resulting in a reduced amount of viral particles and in turn an insufficient presentation of new virus progenies to the immune system. One animal of the nKSCC group was even seronegative, indicating that here tumor formation was probably only caused by UVB exposure alone as found in the two MnPV^-^ controls which also developed tumors. Of note, throughout all groups, VLP-specific antibody responses in final sera correlated well with their neutralizing capacity in pseudovirion-based neutralization assays (Fig 6B). These results support the notion of a still functioning humoral immune surveillance during chronic UV exposure.

Host cell dedifferentiation can interfere with replicating episomal DNA and favor its integration, as known for high-risk α-HPVs [86,87]. However, integrated cutaneous HPVs were never reported [68], implying other selection mechanisms that allow growth advantage through genomic driver mutations, making PVs finally dispensable for the maintenance of a malignant phenotype. This was demonstrated by spatial microdissection of a nKSCC (Fig 5B), harboring mutated p53 (R266C) that was completely inactive in the transactivation assay (Fig 10B and 10C).

In any case, cancer has to be considered an evolutionary process [88] in which MnPV is apparently contributing to the first steps of tumor initiation by enhancing the probability for tumor formation, as can be concluded when the outcome of UVB irradiation on infected and uninfected animals is compared (Fig 1A).

Considering the mechanism of DNA damage in general, however, normal cells either undergo a p53-dependent cell cycle arrest that allows DNA repair or they are eliminated by apoptosis [89]. However, certain cutaneous HPVs developed ways to circumvent physiological DNA-damage responses, either by inactivating HIPK2-mediated phosphorylation of p53 and subsequent apoptosis [25] or by targeting the pro-apoptotic protein Bak for degradation [48,90]. In combination with a delay of DNA repair mechanisms such as excision of UV-induced CPDs (Fig 7A) [26] or homology dependent repair [29], the accumulation and proliferation of UV-damaged stem-like cancer cells can be favored [91].

As already reported more than a decade ago, HPV5 and HPV8 can diminish the excision repair of UV-induced cyclobutane pyrimidine dimers (CPDs) [26], which can lead to the generation of DNA double-strand breaks (DSBs) by DNA replication fork collapse during S-phase [52]. In fact, this previously described mechanism leads to an attenuated CPD repair and persisting γH2AX foci in HPV5 and HPV8 E6 positive human keratinocytes *in vitro* and in epithelial cells after UV irradiation of HPV8 E6 transgenic mice [33]. Although UV irradiation itself also induces γH2AX foci [92], they disappear after repair, allowing the cells to proliferate again [55]. In wounded skin of HPV8-E6 transgenic mice, γH2AX foci could also be observed, probably as a result of reactive oxygen species that appear during healing and interfere with transcription or activity of DNA repair enzymes [50].

In line with this assumption is the finding that *in vitro* MnPV E6/E7 expression attenuated CPD repair in *Mastomys coucha* keratinocytes (Fig 7A). This may have led to an enhanced number of DSBs and in turn γH2AX foci in E6/E7-expressing keratinocytes when compared to E6/E7-negative cells (Fig 7B) which disappear more slowly. Repair kinetics apparently differs with respect to previous studies [26,93–95] which can, however, be attributed to different experimental settings, cell types and UV sources and doses. Nevertheless, comparing irradiated skin of MnPV^-^ and MnPV^+^ animals (Fig 7D), the same scenario as described above could be discerned, suggesting that—similar to human cutaneous HPV types—MnPV can interfere with DNA damage responses both under *in vitro* and *in vivo* conditions.

Notably, MnPV apparently can induce γH2AX foci even without additional UVB exposure (Figs 7E and 8). Histone H2AX can be phosphorylated upon genotoxic stress by upstream kinases such as ATM/ATR along with the DNA-dependent protein kinase (DNA-PK) [53]. In UV-induced KSCCs with high viral loads, ISH signals and γH2AX staining coincided, mostly in suprabasal layers where substantial viral replication is taking place [70]. In contrast, nKSCCs with low amounts of viral DNA were negative for both signals because these tumors lack the capacity to differentiate (Fig 11). This may reflect an ATM-dependent DNA damage response in such lesions, known to be essential for viral DNA amplification in differentiated cells [96,97]. How a permissive environment for MnPV with early and late transcription is altered during dedifferentiation where the viral DNA is lost, is currently unknown and awaits further elucidation.

Consistent with many human cutaneous HPVs [24], but in contrast to HPV16 E6, MnPV E6 is not degrading p53 and can only marginally affect its transactivating function (Fig 9A and 9B). Based at least on this experimental read-out, MnPV may affect other pathways that account for enhanced tumor incidence when compared to virus-free animals (Fig 1B). Notably, a complete novel view about the oncogenic potential of “high-risk” cutaneous HPVs has recently been reported for HPV8, a skin type involved in the development of the rare hereditary disease *Epidermodysplasia verruciformis* (EV) [98]. Here, the E6 protein has been shown to down-regulate the microRNA-203 that both interferes with cell differentiation and up-regulates ΔNp63, another member of the p53 family. This finding reasonably explains the potential of HPV8 to hinder differentiation and in turn its capacity to stimulate proliferation of undifferentiated cells [99].

UV as an environmental carcinogen damages the DNA by forming photoproducts that mainly lead to C→T and CC→TT transitions at sites of neighboring pyrimidines, therefore known as UV signatures [22]. Considering the mutational landscape, *TP53* is the most frequently mutated gene in human SCCs [100]. Likewise, sequence analysis of *Mastomys coucha Trp53* also showed mutations in 79% of all UV-induced SCCs. Setting the frequency of *Trp53* mutations into a histopathological context, more mutations were found in nKSCCs than in KSCCs (Fig 10B and 10C). Analyzed mutations resulted either in a loss of transactivation activity (Fig 10E), a reduced intracellular half-life (Fig 10F) or both. The two hot-spots R266 and P271 within the DNA-binding domain (Fig 10A) correspond to positions R273 and P278 in human cancer in general (R273) and cutaneous SCCs in particular (P278) [101]. Reconstitution of the intracellular half-life by proteasome inhibition did not restore the transactivation capability of mutants P271F and P271S, arguing against an inverse correlation between the amount of p53 and its ability to transactivate p53-responsive reporter genes (Fig 10G and 10H). Although still active in the reporter assay and therefore probably not contributing to carcinogenesis by a loss of transactivation activity, P145L also displayed a reduced half-life, probably due to the substitution of proline and subsequent enhanced degradation of the misfolded protein [102]. Indeed, similar to human SCCs [100], *Mastomys* also developed SCCs that still carried wildtype *Trp53* (Fig 5, left and middle panel), but additional potential driver mutations may have substituted this function.

Although *TP53* is the most frequently mutated gene in human SCCs, also other potential driver mutations have been identified [100,103]. In the context of the tumors developed in *Mastomys coucha*, we focused our attention on RAS-family members, since UV predominantly induces C→T and CC→TT transitions [22] that are frequently found at hot spot positions in codon 12 of the *HRAS* and *KRAS* genes [104]. However, as far as KSCCs and nKSCCs were tested, irrespective of the *Trp53* status, no activating mutations in *H*, *K or Nras* (codons 12, 13 and 61) could be detected (S3 Table). Nonetheless, taking *Trp53* as a surrogate genome sequence, it is obvious that cells in nKSCCs generally acquired more genomic mutations than cells in KSCCs. Nonetheless, it is reasonable to assume that loss of p53 function is predisposing cells to skin carcinogenesis, since a high mutation frequency could already be detected in pre-malignant actinic keratoses [105]. Islands of hyperproliferative squamous cells with elevated p53 could already be found in UV-irradiated skin (S5B Fig), indicating that those patches may represent an early event in skin carcinogenesis. However, the time frame and temporal order of events favoring KSCC and nKSCC development in our model are unknown and currently under investigation.

Loss of functional epidermal *Trp53* in mice leads to the development of poorly differentiated SCCs, supporting a decisive role of mutated p53 on the phenotype of squamous cells and invasion [62]. Recently, Tovy *et al*. showed that p53 deficiency in mouse embryonic stem cells leads to deregulated DNA methylation patterns, increases phenotypical heterogeneity of these cells and interferes with their differentiation [106]. Early functional loss of epidermal p53 may also account for the histological diversity of the tumor types in our system, as recently shown in a knock-out mouse model [107]. Therefore, loss of functional p53 in a nKSCC harboring p53 mutant R266C (Fig 11) may favor dedifferentiation [64] which could explain the differences in viral load between KSCCs and nKSCCs.

**Fig 12:**
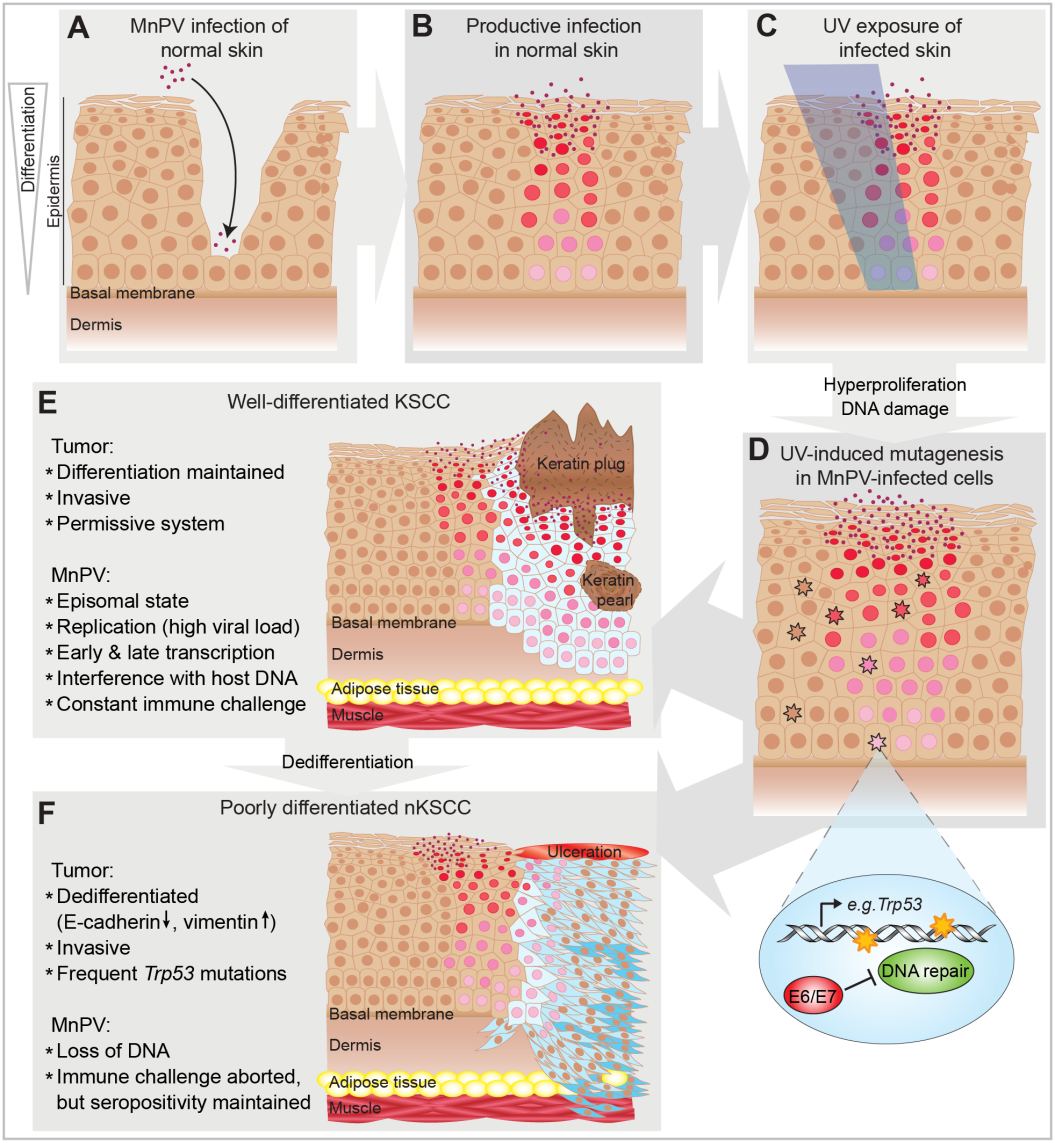
Schematic overview of the mechanism suggested for UV-induced NMSC development in *Mastomys coucha*. **A)** MnPV infects basal epithelial cells of the skin of young animals via small injuries. **B)** MnPV genome is amplified in stratified skin layers (pink and red nuclei) and new virions are released. **C)** UVB irradiation of the skin. **D)** UVB-irradiated skin is hyperproliferative, favoring viral replication and virion formation. UVB-induced photoproducts, e.g. in *Trp53*, occur in keratinocytes (altered nuclei). In uninfected cells, damages are repaired. In infected cells, MnPV-E6/E7 reduce chromosomal stability and inhibit DNA repair. Mutations can accumulate and altered cells become neoplastic. **E)** Neoplastic squamous cells (light blue) start forming a well-differentiated keratinizing SCC, still representing a permissive system that allows viral replication and formation of virions. **F)** When neoplastic squamous cells accumulate further mutations (dark blue), a spindle cell phenotype is acquired, forming a poorly differentiated SCC that may become ulcerated. MnPV cannot replicate in dedifferentiated cells and the viral DNA is subsequently lost.

In conclusion, this is the first study showing cooperation between cutaneous PV infection and UVB radiation in SCC formation in naturally infected animals. It can be considered as a paradigm for a frequent cancer initiated by a cutaneous papillomavirus which is finally no longer required to maintain the malignant state (Fig 12), a situation commonly found in SCCs from patients. With this preclinical model, we are currently investigating the role of skin papillomaviruses at different stages during NMSC development.

## Materials and methods

### Ethics statement

The animal facility of the German Cancer Research Center has been officially approved by responsible authority (Regional Council of Karlsruhe, Schlossplatz 4–6, 76131 Karlsruhe, Germany). The official approval file number is Az 35–9185.64BH DKFZ. Housing conditions are thus in accordance with the German Animal Welfare Act (TierSchG) and EU Directive 2010/63/EU. Regular inspections of the facility are conducted by the Veterinary Authority of Heidelberg (Bergheimer Str. 69, 69115 Heidelberg, Germany). All experiments were in accordance with the institutional guidelines (designated veterinarian according to article 25 of Directive 2010/63/EU and Animal-Welfare Body according to article 27 of Directive 2010/63/EU) and were officially approved by Regional Council of Karlsruhe (File No G26/12).

### Animals

*Mastomys coucha* from the DKFZ colonies were maintained under SFP conditions in individually ventilated cages (Tecniplast GR900) or in type 3 cages in positive pressure isolators on aspen bedding with curled wood wool as environmental enrichment. Mastomys were constantly kept in a light/dark cycle of 14/10h, an average temperature of 22+/-2°C and a humidity of 55+/-10% according to Directive 2010/63/EU, appendix III and the German legislation. Mastomys were fed ad libitum (Mouse and Rat Maintenance No. 3437, KLIBA NAFAG, Kaiseraugst, Switzerland) and had unlimited access to autoclaved water.

Virus-free animals were obtained by hysterectomies of pregnant *Mastomys coucha* under sterile conditions and kept in a specific pathogen free isolator unit with positive air pressure at the DKFZ [21]. To confirm the virus-free status of the animals, sera and skin samples are regularly tested by ELISA (E2 and L1) and PCR, respectively.

### UV irradiation of animals

Anesthetized animals (3% isoflurane) were irradiated three times per week with UVB at the shaved back in Bio-Spectra cabinets (Vilber Lourmat, Eberhardzell, Germany) with an energy output of 312 nm (UVB) until desired doses were reached. As an example, for achieving a dose of 450 mJ/cm^2^, the irradiation time was about 2 min. During irradiation, the animals were covered with a lid with windows of 2x3 cm to only expose the shaved area of the back. Fourteen weeks-old animals were irradiated with a starting dose of 150 mJ/cm^2^ which was increased weekly by 50 mJ/cm^2^ [32]. To investigate the effect of UVB on tumor development, three groups were established (with final doses of 450, 600 and 800 mJ/cm^2^, respectively). The irradiation was then pursued with the final doses until the animals had to be sacrificed. Animals were checked weekly for the appearance of tumors.

### Immunohistochemistry (IHC)

Skin biopsies and tumors were cut longitudinally with scalpels and fixed in 4% buffered paraformaldehyde, embedded in paraffin, sliced in one to four μm thick sections and either stained by H&E or used for IHC with the following primary antibodies: anti-Cytokeratin, pan-specific (C-11) (1:100; F3418; Sigma-Aldrich, St. Louis, Missouri, USA), anti-p53 Pab240 (1:30; sc-99; Santa Cruz Biotechnology, Dallas, Texas, USA), anti-Ki-67 (1:200; IHC-00375; Bethyl Laboratories, Montgomery, Texas, USA), anti-CPD TDM-2 (1:600; NMDND001; Cosmo Bio, Carlsbad, California, USA), anti-γH2AX (1:550; MABE205; EMD Millipore, Billerica, Massachusetts, USA) and anti-Collagen IV (1:50; CL50451AP; Cedarlane, Burlington, Canada). Antigen retrieval was achieved after deparaffinization by heating of the sections for 15–30 min in citrate buffer (pH 6.0) for pan-Cytokeratin, Ki-67, CPDs and p53 or in EDTA (pH 9.0) for γH2AX. For collagen IV staining, the sections were treated for 5 min with 0.05% trypsin. Endogenous peroxidases were blocked with Dako REAL peroxidase blocking solution (Agilent Technologies, Hamburg, Germany) followed by blocking with the Avidin/Biotin Blocking Kit (Linaris Biologische Produkte, Dossenheim, Germany) for 10 min per solution and with 1% BSA/10% goat serum/PBS for 1 h. Between each step, sections were washed for 1 min in PBST (0.5% Tween-20 in PBS). Primary antibodies were diluted in 1% BSA/5% goat serum/PBS and applied overnight at 4°C in a wet chamber. Ki-67 was further detected with the Alkaline Phosphatase/Anti-Alkaline Phosphatase Method as described before [108]. For detection of E-Cadherin and vimentin, the sections were subsequently washed three times for 5 min with PBST, incubated for 45 min with AlexaFluor-594 goat anti-rabbit IgG or AlexaFluor-488 goat anti-mouse IgG in 1% BSA/5% goat serum/PBS (1:1,000; Invitrogen, Carlsbad, California, USA) and washed again three times with PBST. Nuclei were stained for 10 min with DAPI (0.3 μg/ml in PBS) prior to four washes for 5 min in PBS. The other antigens were detected with the Dako REAL Detection System, Peroxidase/AEC, Rabbit/Mouse: Biotinylated secondary antibodies were applied for 20 min followed by washing and incubation with streptavidin-peroxidase for 20 min. After washing AEC/H_2_O_2_ substrate solution (Sigma) or SignalStain DAB Substrate (Cell Signalling Technology, Danvers, Massachusetts, USA) was added. The color reaction was stopped with distilled water followed by counterstaining with hemalum solution (Carl Roth, Karlsruhe, Germany). Sections were mounted with Dako Faramount Aqueous Mounting Medium, covered and imaged with a Keyence BZ-9000 Microscope.

### *In situ* hybridization (ISH)

Tissue sections were hybridized with a biotinylated full-length MnPV-probe using the Biotin-Nick-Translation-Mix (Roche, Mannheim, Germany). A biotinylated pBR322 probe served as negative control. Probes were detected with streptavidin-conjugated HRP (HRP-SA) included in the Tyramide Signal Amplification Kit (PerkinElmer, Waltham, Massachusetts, USA) as described elsewhere [15] with the following changes: sections were cooked for 10 min in citrate buffer (pH 6.0) in a steam pot and digested with proteinase K (2 μg/ml in 0.05 Tris/HCl pH 7.5 at 37°C for 12 min). Endogenous peroxidases were blocked for 10 min with 3% H_2_O_2_ in Tris-buffered saline (TBS). The pre-hybridization mix was applied for 3 h at room temperature. Sections were incubated with the hybridization mix (includes 300 ng/ml probe) at 42°C for 16 h and then washed on a magnetic stirrer for 10 min in 2x SSC (42°C), 1x SSC (room temperature) and 0.5x SSC (room temperature) before blocking with 20% goat serum/25% TNB buffer in TBS for 45 min. The sections were incubated for 30 min with HRP-SA (1:250 in 10% goat serum/25% TNB buffer in TBS) and washed three times for 3 min in TBS. The signal was amplified by an incubation with biotinyl tyramide (1:50 in Amplification Diluent) for 20 min followed by a second incubation with HRP-SA and three washes. Staining with the AEC staining kit (Sigma-Aldrich) and following steps were performed as described for IHC.

### Preparation of nucleic acids from tissue

Sacrificed shaved animals were shock-frozen in liquid nitrogen and the epidermal layer was scratched to a powder with a scalpel and collected onto aluminum foils placed on dry ice to keep the cold chain. The obtained skin powder was transferred to pre-cooled reaction tubes and stored at -80°C. From tumors, thin slices were cut with a scalpel and also deep frozen. To avoid cross contamination, surgical instruments and aluminum foils were changed after every single sample. The DNA was extracted as described elsewhere [17]. RNA was isolated and reverse transcribed as previously described [51]. To extract genomic DNA from specified areas of SCCs, one to two 3 μm-thick sections were deparaffinized. Microdissection was performed using an EVOS Core Cell Imaging System and a cannula. Dissected groups of cells were transferred into a reaction tube and lysed overnight in a ThermoMixer (Eppendorf, Hamburg, Germany) at 56°C and 600 rpm in 25 μl Chelex 100 Resin (5% w/v suspended in water) (Bio-Rad, Hercules, California, USA) and 5 μg Proteinase K (Gerbu, Heidelberg, Germany). The suspension was vortexed for 10 sec, boiled for 8 min and centrifuged for 3 min at 12,000 g to pellet the Chelex resin. Two to five μl of the supernatant were used as a template for the qPCR.

### RT-PCR

*GAPDH*, *Trp53*, *H/K/Nras* and the viral *E1^E4*, *E6*, *E7 and L1* cDNAs were amplified by PCR using PRECISOR High-Fidelity DNA Polymerase (BioCat, Heidelberg, Germany) and appropriate forward and reverse primers (see S4 Table for primer summary) from 20–50 ng of reverse transcribed RNA according to the manufacturer’s protocol. Thermal cycling conditions for PCRs were based on a primary denaturation step at 98°C for 2 min, followed by 26–37 cycles of 30 sec at 98°C, 20 sec at 57–60°C, 30 sec at 72°C and a final extension step of 5 min at 72°C. DNA fragments were separated by agarose gel electrophoresis, stained and visualized by UV light. For sequencing of *Trp53* and *H/K/Nras* cDNAs, PCR products were extracted from agarose gels with the QIAquick Gel Extraction Kit (Qiagen, Hilden, Germany) and sequenced with appropriate primers (see S4 Table) by the GATC Biotech Sanger Service (GATC Biotech, Konstanz, Germany). Chromatograms were analyzed with Chromas 2.5.3 (Technelysium, South Brisbane, Australia). *Trp53* mutations were detected by alignment of wildtype *Mastomys Trp53* cDNA with cDNA obtained from tumors.

### Detection of MnPV status in animal samples by Southern blot hybridization

Extracted DNA was digested with ApaI, XbaI or XhoI as indicated in the figure legend. Four μg DNA were digested for 8 h at 37°C prior to electrophoretic size separation of fragments in a 0.8% agarose gel. The DNA was blotted overnight onto a GeneScreen Plus Hybridization Transfer Membrane (PerkinElmer). The filters were hybridized with a ^32^P-dCTP labeled unit-length MnPV DNA as previously described [16].

### Quantitative PCR

Quantification of MnPV DNA was performed as previously described with 50 ng of total DNA, the iTaq Universal SYBR Green Supermix (Bio-Rad, Hercules, California, USA) and forward/reverse primers for the MnPV-L1 gene and the single-copy-number gene β-Globin to determine the number of input cell equivalents (see S4 Table) [21]. MnPV DNA copy numbers were determined in duplicate by using standard curves generated in the same PCR run with a standard containing MnPV and β-globin plasmids. MnPV DNA load was defined as the number of MnPV genomes per two β-globin copies [109]. Sensitivity of the method was 5 MnPV genomes per sample and quantification was linear from 5 to 5 × 10^8^ MnPV copies. For population comparisons, all samples from animals of the MnPV^+^ colony were grouped according to their tissue type.

### Serological analyses

Blood was collected in a 1.5 ml reaction tube after puncture of the submandibular vein and incubated until it was clotted. After centrifugation at 6200 g the serum was transferred into a fresh reaction tube and stored at -20°C. Seroresponses against MnPV capsids were measured with a VLP-ELISA as described elsewhere [21].

### Cloning of p53 expression vectors

The *Mastomys coucha* wildtype p53 (p53wt) coding sequence was amplified from cDNA obtained from freshly isolated keratinocytes and cloned into the pPK-CMV-E3 expression vector (PromoKine, Heidelberg, Germany) enabling expression of proteins tagged to HA [51]. Mutants of p53 (P145L, R266C, P271F and P271S) were produced by site-directed mutagenesis of the pPK-p53wt vector using appropriate forward and reverse primers (see S4 Table). In 25 μl reactions 200 ng of template plasmid were amplified with 1.25 U Pfu DNA polymerase (Thermo Fisher Scientific, Waltham, Massachusetts, USA), 2 μM mutagenesis primer pair and 400 μM dNTPs. The reaction mixture was heated to 95°C for 2 min followed by 18 cycles of 95°C for 30 s, 60°C for 50 s and 68°C for 10 min and one step at 68°C for 7 min. The template vector was digested with 10 U DpnI (New England Biolabs, Frankfurt am Main, Germany) for 1.5 h at 37°C. Chemically competent *E*. *coli* were transformed with 5 μl of the reaction mix. The desired mutation was verified by sequencing of plasmids obtained from single bacterial clones.

### Cell culture and transactivation reporter assay

The functionality of *Mastomys coucha* wildtype and mutant p53 was tested in a transactivation assay as described before [51]. Briefly, H1299 cells (a kind gift from T. Hofmann, DKFZ) lacking endogenous p53 were co-transfected with pPK-p53 expression plasmid, pG13-luc reporter plasmid encoding firefly luciferase under the control of the p53 consensus binding site of the p21 promoter and pRL-TATA encoding a TATA box-driven *Renilla* luciferase for normalization of the signals. To investigate the effect of E6 on p53, cells were additionally transfected with increasing amounts (0, 250, 500 or 750 ng) of MnPV-E6 expression plasmid (pCMV-3tag_MnPV-E6). As a positive control for this setup, cells were transfected with human p53 (in pPK) and increasing amounts (0, 250, 500 or 750 ng) of HPV16-E6 (pCMV-3tag HPV16-E6). Transfections were performed in duplicates either in the absence or presence of 5 μM MG132 (LifeSensors, Malvern, Pennsylvania, USA). Twenty-four hours after transfection, samples were harvested and reporter activity was measured with the Dual-Luciferase Reporter Assay System (Promega, Fitchburg, Wisconsin, USA) according to the manufacturer’s protocol in a Synergy2 reader (BioTek, Bad Friedrichshall, Germany).

### CPD ELISA

Kera5 cells were cultured for various periods after irradiation with 25 mJ/cm^2^ UVB and genomic DNA was isolated using the DNeasy kit (Qiagen). The amounts of CPDs were determined by an ELISA using anti-CDP antibody TDM-2 as previously described [110]. Briefly, harvested DNA was denatured for 10 min at 99°C and chilled on ice for 15 min. Fifty ng/well DNA in 50 μl were loaded to protamine sulfate pre-coated polyvinylchloride microtiter plates and incubated at 37°C to evaporate the liquid. The wells were washed five times with PBS-T (0.05% Tween-20) prior to 30 min incubation with 150 μl/well 2% FCS in PBS at 37°C. The wells were washed again and 70 μl of TDM-2 (1:1,000) were added. After 30 min at 37°C and five washes, 100 μl/well of anti-mouse IgG (1:10,000; W402B, Promega) were added and incubated again for 30 min. After five washings, 100 μl/well substrate solution (10 mg ABTS, 4 μl H_2_O_2_ (35%), 10 ml Citrate-phosphate buffer (pH4.2) were added. The color reaction was measured at 405 nm with a SPECTROstar Nano (BMG LABTECH, Ortenberg, Germany).

### Quantification γH2AX of foci after UV irradiation

Kera5 cells obtained from *Mastomys coucha* skin were obtained and cultured as previously described [51], retrovirally transduced with pLXSN (empty vector or coding for MnPV-E6/E7) [111], selected and checked via RT-PCR for expression as described elsewhere [90]. For UV irradiation, cells grown on glass cover slides were washed once with PBS and irradiated with 50 mJ/cm^2^ of UVB (Waldmann UV181BL (Waldmann, Villingen-Schwenningen, Germany) with an output range of 280–320 nm as measured with a detector (Waldmann Variocontrol). After 7, 24, 48 and 72 h of further incubation, the cells were subsequently washed for 5 min with PBS, fixed for 10 min with 4% PFA, blocked for 1 h with 1% BSA/0.5% Triton X-100 in PBS and incubated with anti-γH2AX antibody (1:650; MABE205; EMD Millipore) for 1 h at room temperature. The cells were washed three times for 5 min in PBS prior to incubation with AlexaFluor-594 goat anti-rabbit IgG (1:1,000; Invitrogen) for 45 min at room temperature. The cells were subsequently washed in PBS, nuclei were stained for 10 min with DAPI (0.3 μg/ml in PBS), washed again four times for 5 min in PBS and mounted with Aqua-Poly/Mount (Polysciences, Hirschberg an der Bergstraße, Germany). Cells were imaged with a Keyence BZ-9000 Fluorescence Microscope and γH2AX foci analysis was performed using a FIJI (ImageJ) [112] macro developed at DKFZ Light Microscopy Core Facility. Shortly, the Find Maxima tool with Segmented Particles above lower threshold option was used for segmentation, and Analyze Particles tool was used for foci scoring.

### Western blotting

H1299 cells were transfected with 1.5 μg of p53 expression plasmids and 250 ng pEGFP-N1 (transfection control) using Lipofectamine3000 according to the manufacturer’s protocol and harvested 18 hours after transfection. To monitor potential differences of p53 stability, transfected cells were treated with 5 μM MG132 8–12 hours *prior to* harvesting. After treatment or transfection cells were collected, washed in PBS and lysed for 30 min on ice in RIPA buffer (20 mM Tris pH 7.5, 150 mM NaCl, 1 mM Na_2_EDTA, 1 mM EGTA, 1% NP-40, 1% sodium deoxycholate) including 1x cOmplete Protease Inhibitor Cocktail (Roche). Extracts obtained from the transactivation assay were used without adding additional inhibitors. Lysates were centrifuged for 30 min at 4°C and 17,000 g and supernatants were quantified using Bio-Rad Protein Assay Dye Reagent Concentrate (Bio-Rad). Thirty μg of denatured cell lysate/lane were loaded to 12% SDS-PAGE. After blotting, proteins were detected with anti-HA (3F10, Roche), anti-GFP (sc-8334, Santa Cruz), anti-FLAG M2 (F3165, Sigma-Aldrich) or anti-mouse-actin antibodies prior to detection with goat anti-mouse-HRP (W402B, Promega) or goat anti-rat-HRP (Jackson ImmunoResearch, Newmarket, UK).

### Alignments

Murine *Trp53* (Gene ID 22059) and human *TP53* (Gene ID 7157) served as reference sequences for alignments with *Mastomys coucha Trp53* (GenBank Accession: KY626317) using Clustal 2.0.12.

### Statistical analysis

Data analyses and graphic representations were performed with GraphPad Prism 5.0 Software and the respective statistical test indicated in the figure legends at 95% confidence interval and a p-value of 0.05 to assess significance.

## Supporting information

S1 Fig Kaplan-Meier curves for the tumor incidence in the different dose groups. Kaplan-Meier curves depicting the percentage of irradiated virus-infected (MnPV^+^, UV^+^) and virus-free (MnPV^-^, UV^+^) tumor-bearing animals divided by dose groups. The legend indicates the final UVB doses respectively (Mantel-Cox test; MnPV^+^: all differences ^ns^p>0.05; MnPV^-^: not assessable).
(TIF)

S2 Fig H&E stainings of a non-UV tumor (A) and a UV-induced KSCC in MnPV^+^ animals (B). Both entities are composed of well-differentiated hyperproliferative atypical squamous cells. Higher magnifications reveal koilocytes [enlarged, crenated nuclei with perinuclear halos] (insets, arrows) indicative for papillomavirus infection (d: dermis; e: epidermis; f: fat; k: keratin; scale bars: overviews: 1 mm, insets: 100 μm).
(TIF)

S3 Fig Progression of a keratinized lesion towards a partially non-keratinized lesion. Over time, a lesion which first was keratinizing (white arrows) progressed to a tumor that partly looked like an nKSCC (blue arrow) and partly like a KSCC.
(TIF)

S4 Fig IHC stainings for Ki-67 in KSCC and nKSCC. Areas shown correspond to the insets in Fig 5. **(A)** KSCC. **(B)** nKSCC. (Scale bars: 100 μm).
(TIF)

S5 Fig IHC stainings for p53 and pan-cytokeratin reveal elevated p53 levels in invading squamous cells. **A)** Unirradiated skin without detectable p53 signals. **B)** Islands of basal keratinocytes show strong nuclear p53 signals (blue arrows) in UV-irradiated, hyperproliferative epidermis in a MnPV^+^ animal. **C)** Altered squamous cells migrating out of the epidermis (black arrow) show strong p53 staining (blue arrow) **(D)** in an nKSCC (Scale bars: 100 μm).
(TIF)

S1 Table Quantification of viral loads related to Fig 4A. (PDF)

S2 Table Viral loads corresponding to viral transcripts in Fig 4C. (PDF)

S3 Table Summarized sequencing results of *Hras*, *Kras* and *Nras* cDNAs of SCCs. (PDF)

S4 Table Overview and summary of primers used in this study. (PDF)
