# Supplementary figures and images for: The interplay of UV and cutaneous papillomavirus infection in skin cancer development
Source: PLoS Pathog. 2017 Nov 30;13(11):e1006723. doi: 10.1371/journal.ppat.1006723 (PMC5708609; doi:10.1371/journal.ppat.1006723)

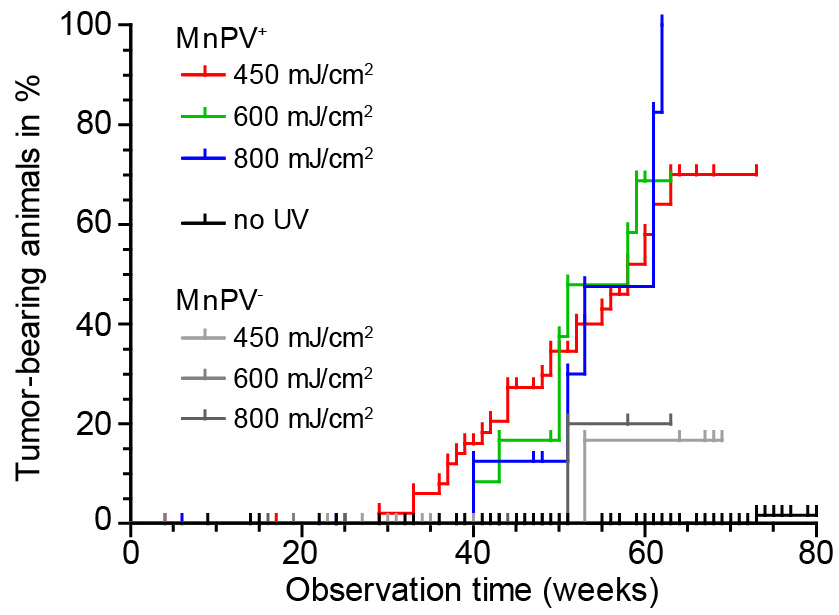

Supplement: S1 Fig — Kaplan-Meier curves depicting the percentage of irradiated virus-infected (MnPV+, UV+) and virus-free (MnPV-, UV+) tumor-bearing animals divided by dose groups. The legend indicates the final UVB doses respectively (Mantel-Cox test; MnPV+: all differences nsp>0.05; MnPV-: not assessable). (TIF) [file ppat.1006723.s001.tif]

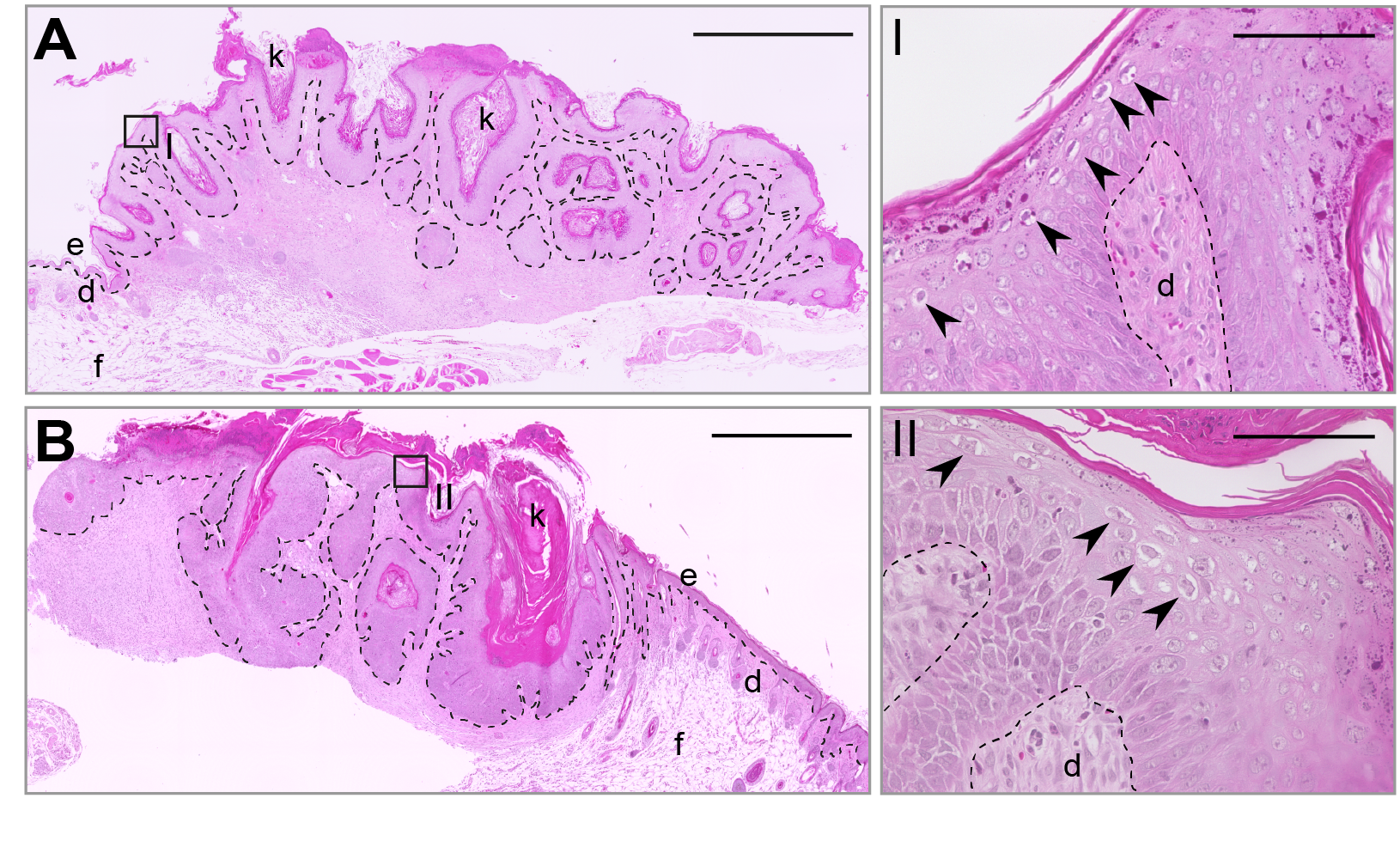

Supplement: S2 Fig — Both entities are composed of well-differentiated hyperproliferative atypical squamous cells. Higher magnifications reveal koilocytes [enlarged, crenated nuclei with perinuclear halos] (insets, arrows) indicative for papillomavirus infection (d: dermis; e: epidermis; f: fat; k: keratin; scale bars: overviews: 1 mm, insets: 100 μm). (TIF) [file ppat.1006723.s002.tif]

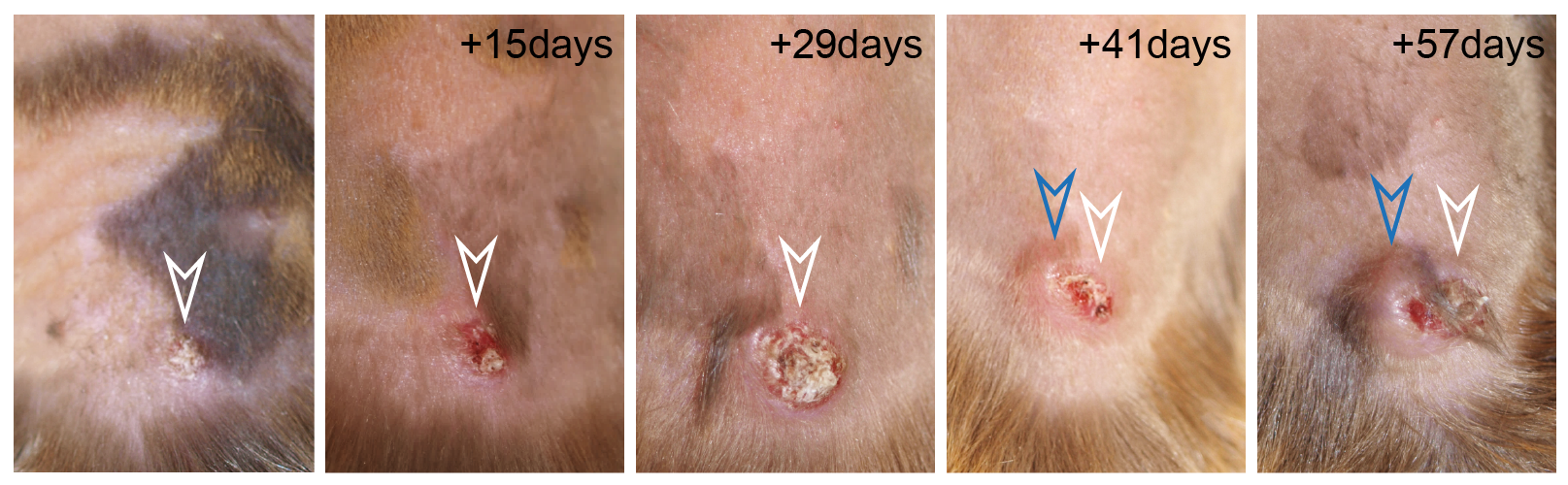

Supplement: S3 Fig — Over time, a lesion which first was keratinizing (white arrows) progressed to a tumor that partly looked like an nKSCC (blue arrow) and partly like a KSCC. (TIF) [file ppat.1006723.s003.tif]

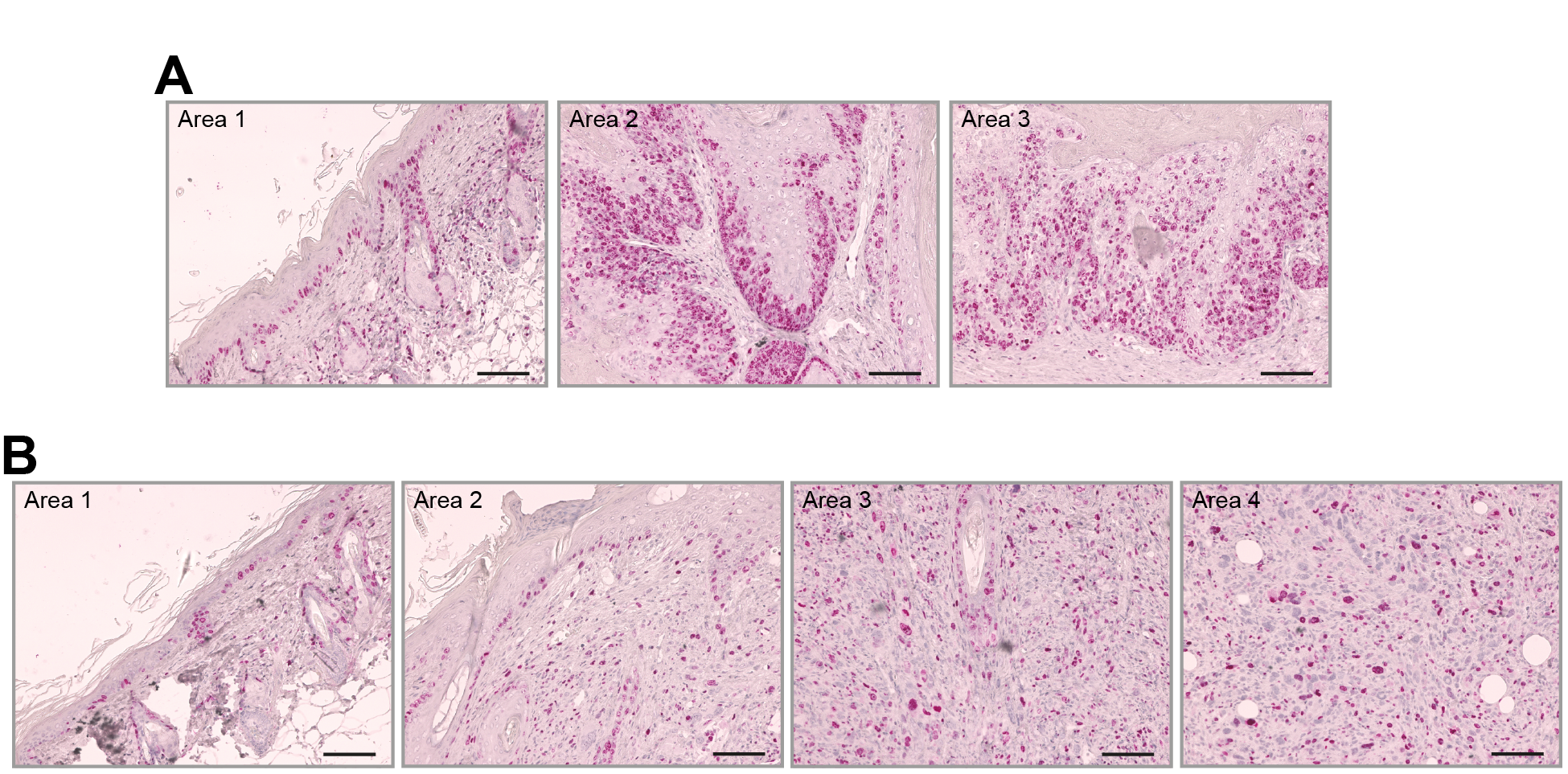

Supplement: S4 Fig — Areas shown correspond to the insets in Fig 5. (A) KSCC. (B) nKSCC. (Scale bars: 100 μm). (TIF) [file ppat.1006723.s004.tif]

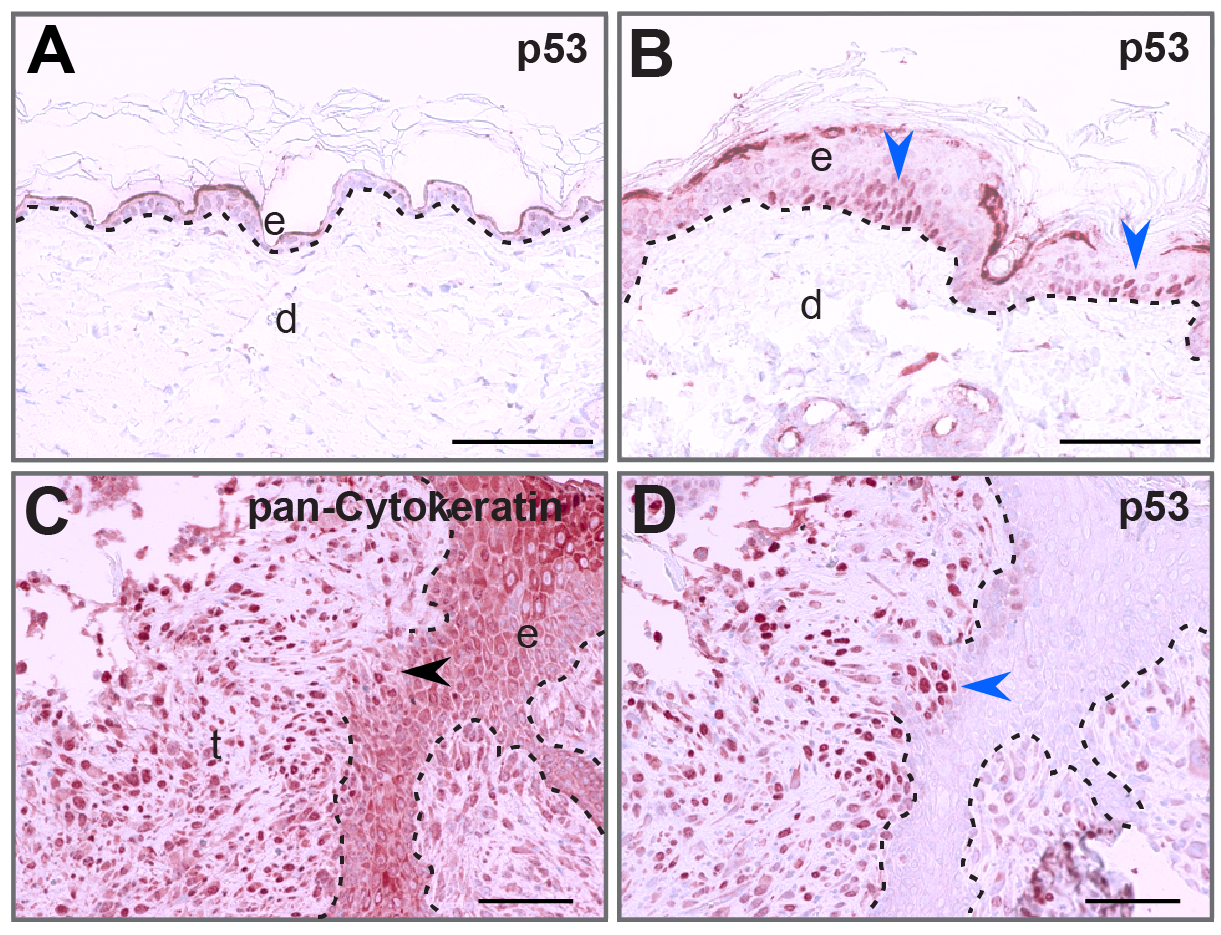

Supplement: S5 Fig — A) Unirradiated skin without detectable p53 signals. B) Islands of basal keratinocytes show strong nuclear p53 signals (blue arrows) in UV-irradiated, hyperproliferative epidermis in a MnPV+ animal. C) Altered squamous cells migrating out of the epidermis (black arrow) show strong p53 staining (blue arrow) (D) in an nKSCC (Scale bars: 100 μm). (TIF) [file ppat.1006723.s005.tif]
