# Supplementary material for: The interplay of UV and cutaneous papillomavirus infection in skin cancer development
Source: PLoS Pathog. 2017 Nov 30;13(11):e1006723. doi: 10.1371/journal.ppat.1006723 (PMC5708609; doi:10.1371/journal.ppat.1006723)
Supplement: S1 Table — (PDF) [file ppat.1006723.s006.pdf]

S1 Table. Quantification of viral loads related to Fig 4A.

Colony	Animal	Sample	Range genomes/cell	Median genomes/cell
$MnPV^+$	UV^-	ctrl skin	0.0021 – 2.3873	0.0329
	UV^+	ui skin	0.0008 – 89.1251	0.0977
		UV skin	0.0023 – 7709.0347	0.1552
		nKSCC	0.0006 – 1.5653	0.0200
		KSCC	0.0208 – 33265.9553	736.2071
		non-UV tumor	5.9938 – 102329.2992	11614.4861
