# Supplementary material for: The interplay of UV and cutaneous papillomavirus infection in skin cancer development
Source: PLoS Pathog. 2017 Nov 30;13(11):e1006723. doi: 10.1371/journal.ppat.1006723 (PMC5708609; doi:10.1371/journal.ppat.1006723)
Supplement: S2 Table — (PDF) [file ppat.1006723.s007.pdf]

**S2 Table. Viral loads corresponding to viral transcripts in Fig 4C.**

| Tumor | Sample | MnPV genomes/cell |
|---|---|---|
| Non-UV tumor | nUV1 | 8261,085 ± 544,020 |
| | nUV2 | 12065,071 ± 1119,243 |
| | nUV3 | 63,907 ± 19,569 |
| KSCC | K1 | 390,166 ± 22,375 |
| | K2 | 14718,994 ± 1041,865 |
| nKSCC | nK1 | 0,0040 ± 0,0003 |
| | nK2 | 0,0006 ± 0,0001 |
| | nK3 | 0,0059 ± 0,0011 |
| | nK4 | 1,565 ± 0,175 |
