# Supplementary material for: The interplay of UV and cutaneous papillomavirus infection in skin cancer development
Source: PLoS Pathog. 2017 Nov 30;13(11):e1006723. doi: 10.1371/journal.ppat.1006723 (PMC5708609; doi:10.1371/journal.ppat.1006723)
Supplement: S3 Table — (PDF) [file ppat.1006723.s008.pdf]

S3 Table. Summarized sequencing results of H/K/N*ras* cDNAs of SCCs.

Animal	Tumor	H-*ras* status	N-*ras* status	K-*ras* status	p53 status	Tumor type
#1	T1	wildtype	wildtype	wildtype	mutated	mixed
#2	T1	codon 71 C>T (silent)	wildtype	wildtype	mutated	nKSCC
	T2	wildtype	wildtype	wildtype	mutated	KSCC
#3	T1	wildtype	wildtype	not tested	mutated	KSCC
#4	T1	wildtype	wildtype	wildtype	mutated	nKSCC
#5	T1	wildtype	wildtype	wildtype	mutated	nKSCC
#6	T1	wildtype	wildtype	wildtype	mutated	nKSCC
#7	T1	wildtype	wildtype	wildtype	mutated	KSCC
#8	T1	wildtype	wildtype	wildtype	mutated	nKSCC
#9	T1	wildtype	wildtype	wildtype	wildtype	KSCC
	T2	wildtype	wildtype	wildtype	wildtype	KSCC
	T3	wildtype	not tested	wildtype	wildtype	KSCC
#10	T1	wildtype	wildtype	wildtype	wildtype	nKSCC
		**n = 13**	**n = 10**	**n = 12**		
