# Supplementary material for: The interplay of UV and cutaneous papillomavirus infection in skin cancer development
Source: PLoS Pathog. 2017 Nov 30;13(11):e1006723. doi: 10.1371/journal.ppat.1006723 (PMC5708609; doi:10.1371/journal.ppat.1006723)
Supplement: S4 Table — (PDF) [file ppat.1006723.s009.pdf]

S4 Table: Primers used in this study.

RT-PCR

**MnPV E1^E4 (58°C)**	
for	5′- TGAAGAAGCTCTACACCGCA-3′
rev	5′- GTCTCCTCCTTTCGGGTGC-3′
**MnPV E6 (57°C)**	
for	5′- CGAGGTTTCTTACCCAGGAGG-3′
rev	5′- TGCAAATTCTGCACCGTGC-3′
**MnPV E7 (57°C)**	
for	5′- ATGATAGGACCTGACACCACGC-3′
rev	5′- CTTCAAGATCTTCCATTC-3′
**MnPV L1 (57°C)**	
for	5′- TCTACACCCGTCATTGTCCA-3′
rev	5′- GCCACGAGCTATCTCCACTC-3′
**GAPDH (60°C)**	
for	5′-CTTCATTGACCTCAACTACATGGTC-3′
rev	5′-GCAGTGATGGCATGGACTGTG-3′
**Trp53 (57°C)**	
for	5′- TGACAGCCATGGAGTATTCGG -3′
rev	5′- CTGAGTCAGGCCCCACTTTC -3′
**H-ras (53°C)**	
for	5′-ATGACAGAATACAAGCTTGTGGTG-3′
rev	5′-CTGTACTGATGGATGTCCTCGAAG-3′
**K-ras (57°C)**	
for	5′-TGCTGAAAATGACTGAGTATAAACTTG-3′
rev	5′-ATAATGGTGAATATCTTCAAATGATTTAG-3′
**N-ras (57°C)**	
for	5′-GGTGTGAAATGACTGAGTACAAACTG-3′
rev	5′-CCTGTAGAGGTTAATATCTGCAAATG-3′

Sequencing of amplified cDNA

**Trp53**	
for	5′-TATGAGCCACCCGAGG-3′
rev	5′-GTCTTCCAGTGTGATGATGG-3′
**H-ras**	
for	5′-ATGACAGAATACAAGCTTGTGGTG-3′
rev	5′-CTGTACTGATGGATGTCCTCGAAG-3′
**K-ras**	
for	5′-TGCTGAAAATGACTGAGTATAAACTTG-3′
rev	5′-ATAATGGTGAATATCTTCAAATGATTTAG-3′
**N-ras**	
for	5′-GGTGTGAAATGACTGAGTACAAACTG-3′
rev	5′-CCTGTAGAGGTTAATATCTGCAAATG-3′

Quantitative PCR

**MnPV L1**	
for	5′- ACGGCAACTCATGCTTCTTC-3′
rev	5′- CTCTGTGCCTGTCCATCCTT-3′
**β-Globin**	
for	5′- ACCATGGTGCACCTTACTGAC-3′
rev	5′- TCCAGGCACCCAACTTCTAC-3′

Cloning of p53 mutants

**P145L**	
for	5′- TCAGCGACACACCTCTAGCTGGGAGCCGG-3′
rev	5′- CCGGCTCCCAGCTAGAGGTGTGTCGCTGA-3′
**R266C**	
for	5′- ACAGCTTTGAAGTTTGCATTTGTGCCTGC-3′
rev	5′- GCAGGCACAAATGCAAACTTCAAAGCTGT-3′
**P271F**	
for	5′- ATTTGTGCCTGCTTTGGGAGAGACCGTC-3′
rev	5′- GACGGTCTCTCCCAAAGCAGGCACAAAT-3′
**P271S**	
for	5′- ATTTGTGCCTGCTCTGGGAGAGACCGTC-3′
rev	5′- GACGGTCTCTCCCAGAGCAGGCACAAAT-3′
